# Publisher Correction: Equivalence and its invalidation between non-Markovian and Markovian spreading dynamics on complex networks

**DOI:** 10.1038/s41467-019-12577-9

**Published:** 2019-10-09

**Authors:** Mi Feng, Shi-Min Cai, Ming Tang, Ying-Cheng Lai

**Affiliations:** 1 School of Mathematical Sciences, Shanghai Key Laboratory of PMMP, East China Normal University, Shanghai, 200241 China; 2 Web Sciences Center, University of Electronic Science and Technology of China, Chengdu, 611731 China; 3 Big Data Research Center, University of Electronic Science and Technology of China, Chengdu, 611731 China; 4 Shanghai Key Laboratory of Multidimensional Information Processing, East China Normal University, Shanghai, 200241 China; 5 School of Electrical, Computer and Energy Engineering, Arizona State University, Tempe, AZ 85287 USA

**Keywords:** Complex networks, Nonlinear phenomena, Phase transitions and critical phenomena

Correction to: *Nature Communications* 10.1038/s41467-019-11763-z, published online 23 August 2019.

The original version of this Article contained errors in multiple equations. As a result of this, the following changes have been made to the originally published version of this Article:

Equation 4 originally incorrectly read:

$${\mathrm{\Psi }}_{{\mathrm{rec}}}\left( \tau \right) = {\smallint }_\tau ^{ + \infty } \psi _{{\mathrm{rec}}}\left( {\tau \prime } \right)d\tau \prime$$

The correct form of Eq. (4) is:

$${\mathrm{\Psi }}_{{\mathrm{inf}}}\left( \kappa \right) = {\smallint }_\kappa ^{ + \infty } \psi _{{\mathrm{inf}}}\left( {\kappa \prime } \right)d\kappa \prime$$

Equation 5 originally incorrectly read:

$${\mathrm{\Psi }}_{{\mathrm{rec}}}\left( \tau \right) = {\smallint }_\tau ^{ + \infty } \psi _{{\mathrm{rec}}}\left( {\tau \prime } \right)d\tau \prime$$

The correct form of Eq. (5) is:

$${\mathrm{\Psi }}_{{\mathrm{rec}}}\left( \tau \right) = e^{ - {\smallint }_0^\tau \omega _{{\mathrm{rec}}}\left( {\tau \prime } \right)d\tau \prime }$$

Equation 6 originally incorrectly read:

$${\mathrm{\Psi }}_{{\mathrm{inf}}}\left( \kappa \right) = {\smallint }_\kappa ^{ + \infty } \psi _{{\mathrm{inf}}}\left( {\kappa \prime } \right)d\kappa \prime$$

The correct form of Eq. (6) is:

$${\mathrm{\Psi }}_{{\mathrm{inf}}}\left( \kappa \right) = e^{ - {\smallint }_0^\kappa \omega _{{\mathrm{inf}}}\left( {\kappa \prime } \right)d\kappa \prime }$$

Equation 12 originally incorrectly read:

$$\left( {\frac{\partial }{{\partial \tau }} + \frac{\partial }{{\partial t}}} \right)S_i\left( {\tau ;t} \right) = - S_i\left( {\tau ,t} \right)\mathop {\sum }\limits_{j = 1}^N a_{ij}{\mathrm{\Phi }}_{i \leftarrow j}\left( {\tau ;t} \right)$$

The correct form of Eq. (12) is:

$$\left( {\frac{\partial }{{\partial \tau }} + \frac{\partial }{{\partial t}}} \right)S_i\left( {\tau ;t} \right) = - S_i\left( {\tau ;t} \right)\mathop {\sum }\limits_{j = 1}^N a_{ij}{\mathrm{\Phi }}_{i \leftarrow j}\left( {\tau ;t} \right)$$

The fourth sentence after Eq. (39) originally incorrectly read: ‘In particular, if both *ϑ*^(1)^(0) and *ϑ*^(*n*)^(0) (*n* ≥ 2) have a finite value, a large value of $\mathop {\sum }\limits_{j = 1}^N a_{ij}\tilde I_j$ will dominate the right-hand side of Eq. (32), making the high-order terms $\mathop {\sum }\limits_0^{ + \infty } \vartheta ^{\left( n \right)}\left( 0 \right)\left( {1/\mathop {\sum }\limits_{j = 1}^N a_{ij}\tilde I_j} \right)^n$ negligibly small.’ The corrected version states instead ‘In particular, if both *ϑ*^(1)^(0) and *ϑ*^(*n*)^(0) (*n* ≥ 2) have a finite value, a large value of $\mathop {\sum }\limits_{j = 1}^N a_{ij}\tilde I_j$ will dominate the right-hand side of Eq. (32), making the high-order terms $\mathop {\sum }\limits_{n = 2}^{ + \infty } \vartheta ^{\left( n \right)}\left( 0 \right)\left( {1/\mathop {\sum }\limits_{j = 1}^N a_{ij}\tilde I_j} \right)^n$ negligibly small.’

This has been corrected in both the PDF and the HTML versions of the Article. Also, the original HTML version of this Article was updated after publication to add links to the Source Data.

