# Supplementary material for: Equivalence and its invalidation between non-Markovian and Markovian spreading dynamics on complex networks
Source: Nat Commun. 2019 Aug 23;10:3748. doi: 10.1038/s41467-019-11763-z (PMC6707263; doi:10.1038/s41467-019-11763-z)
Supplement: Supplementary file 1 — Supplementary Information [file 41467_2019_11763_MOESM1_ESM.pdf]

Supplementary Information for

Equivalence and its invalidation between Non-Markovian and Markovian spreading dynamics on complex networks

Mi Feng, Shimin Cai, Ming Tang, and Ying-Cheng Lai

Corresponding author: Ming Tang (tangminghan007@gmail.com)

SUPPLEMENTARY NOTE 1. STEADY STATE SOLUTION OF NON-MARKOVIAN SIS MODEL WITH TYPE-I ACTIVATION MECHANISM

When the system reaches a steady state, the differential equations governing the evolution of the probability density functions of node i in the infected and susceptible states are, respectively,

$$\frac{d\tilde{I}_i(\tau)}{d\tau} = -\omega_{\text{rec}}(\tau)\tilde{I}_i(\tau) \quad \text{and} \tag{1}$$

$$\frac{d\tilde{S}_i(\tau)}{d\tau} = -\tilde{S}_i(\tau)\sum_{j=1}^{N} a_{ij}\int_0^{+\infty}\omega_{\text{inf}}[\min(\tau,\tau')]\tilde{I}_j(\tau')d\tau', \tag{2}$$

with the initial condition

$$\tilde{I}_i(0) = \tilde{S}_i(0). \tag{3}$$

From Supplementary Eq. (1), we have

$$\tilde{I}_i(\tau) = \tilde{I}_i(0)\Psi_{\text{rec}}(\tau), \tag{4}$$

where $\Psi_{\text{rec}}(\tau) = e^{-\int_0^{\tau}\omega_{\text{rec}}(\tau')d\tau'}$. From Supplementary Eq. (2), we have

$$\tilde{S}_i(\tau) = \tilde{S}_i(0)e^{-\int_0^{\tau}\sum_{j=1}^{N} a_{ij}\int_0^{+\infty}\omega_{\text{inf}}[\min(\tau',\tau'')]\tilde{I}_j(\tau')d\tau' d\tau''}. \tag{5}$$

Substituting, respectively, Supplementary Eqs. (4) and (5) into

$$\tilde{I}_i = \int_0^{+\infty}\tilde{I}_i(\tau)d\tau \quad \text{and} \quad \tilde{S}_i = \int_0^{+\infty}\tilde{S}_i(\tau)d\tau, \tag{6}$$

we obtain the stationary distribution of node i being in the infected and susceptible state, respectively, as

$$\tilde{I}_i = \tilde{I}_i(0)\int_0^{+\infty}\Psi_{\text{rec}}(\tau)d\tau \quad \text{and} \tag{7}$$

$$\tilde{S}_i = \tilde{S}_i(0)\int_0^{+\infty} e^{-\int_0^{\tau}\sum_{j=1}^{N} a_{ij}\int_0^{+\infty}\omega_{\text{inf}}[\min(\tau',\tau'')]\tilde{I}_i(\tau')d\tau' d\tau''}d\tau. \tag{8}$$

Defining the effective recovery rate as

$$\delta_{\text{eff}} = 1/\int_0^{+\infty}\Psi_{\text{rec}}(\tau)d\tau, \tag{9}$$

we can reduce Supplementary Eq. (7) to

$$\tilde{I}_i = \frac{\tilde{I}_i(0)}{\delta_{\text{eff}}}. \tag{10}$$

Combining Supplementary Eqs. (4) and (10), we obtain

$$\tilde{I}_i(\tau) = \tilde{I}_i\delta_{\text{eff}}\Psi_{\text{rec}}(\tau). \tag{11}$$

Equation (8) can then be rewritten as

$$\tilde{S}_i = \tilde{S}_i(0) \int_0^{+\infty} e^{-\delta_{\rm eff} \int_0^{\tau} \int_0^{+\infty} \omega_{\rm inf}[\min(\tau',\tau'')] \Psi_{\rm rec}(\tau') d\tau' d\tau'' \sum_{j=1}^{N} a_{ij} \tilde{I}_j} d\tau. \quad (12)$$

Dividing Supplementary Eq. (10) by Supplementary Eq. (12) and using the initial condition Supplementary Eq. (3), we obtain

$$\delta_{\rm eff} \tilde{I}_i = \frac{\tilde{S}_i}{\int_0^{+\infty} e^{-\delta_{\rm eff} \int_0^{\tau} \int_0^{+\infty} \omega_{\rm inf}[\min(\tau',\tau'')] \Psi_{\rm rec}(\tau') d\tau' d\tau'' \sum_{j=1}^{N} a_{ij} \tilde{I}_j} d\tau}, \quad (13)$$

which can be further reduced to

$$\tilde{I}_i = \frac{\tilde{S}_i}{\delta_{\rm eff} \int_0^{+\infty} e^{-\Omega(\tau) \sum_{j=1}^{N} a_{ij} \tilde{I}_j} d\tau}, \quad (14)$$

where

$$\Omega(\tau) = \delta_{\rm eff} \int_0^{\tau} \int_0^{+\infty} \omega_{\rm inf}[\min(\tau', \tau'')] \Psi_{\rm rec}(\tau') d\tau' d\tau''. \quad (15)$$

Note that $\Omega(0) = 0$. Defining $\vartheta(\tau)$ as the inverse function of $\Omega(\tau)$, we have $\vartheta(0) = 0$. Equation (14) can be rewritten as

$$\tilde{I}_i = \frac{\tilde{S}_i}{\delta_{\rm eff} \int_0^{+\infty} e^{-\xi} d[\vartheta(\frac{\xi}{\sum_{j=1}^{N} a_{ij} \tilde{I}_j})]}, \quad (16)$$

where $\xi = \Omega(\tau) \sum_{j=1}^{N} a_{ij} \tilde{I}_j$. Expanding $\vartheta(\tau)$ as a Taylor series about $\tau = 0$, we have

$$\vartheta(\frac{\xi}{\sum_{j=1}^{N} a_{ij} \tilde{I}_j}) = \vartheta(0) + \sum_{n=1}^{+\infty} \frac{1}{n!} \vartheta^{(n)}(0) (\frac{\xi}{\sum_{j=1}^{N} a_{ij} \tilde{I}_j})^n, \quad (17)$$

where $\vartheta(0) = 0$ and $\vartheta^{(n)}(0)$ is the nth derivative of $\vartheta^{(n)}(\tau)$. Note that, in order to have meaningful solutions, the right side of Supplementary Eq. (17) must converge. Combining Supplementary Eqs. (16) and (17), we obtain

$$\tilde{I}_i = \frac{\tilde{S}_i}{\delta_{\rm eff} \int_0^{+\infty} e^{-\xi} d[\sum_{n=1}^{+\infty} \frac{1}{n!} \vartheta^{(n)}(0) (\frac{\xi}{\sum_{j=1}^{N} a_{ij} \tilde{I}_j})^n]}, \quad (18)$$

which can be rearranged as

$$\tilde{I}_i = \frac{\tilde{S}_i}{\delta_{\rm eff} \sum_{n=1}^{+\infty} \frac{1}{(n-1)!} \vartheta^{(n)}(0) (\frac{1}{\sum_{j=1}^{N} a_{ij} \tilde{I}_j})^n \int_0^{+\infty} \xi^{n-1} e^{-\xi} d\xi}. \quad (19)$$

Using the property of the gamma function

$$\Gamma(n) \equiv \int_0^{+\infty} \xi^{n-1} e^{-\xi} d\xi = (n-1)!, \quad (20)$$

we have

$$\tilde{I}_i = \frac{\tilde{S}_i}{\delta_{\text{eff}} \sum_{n=1}^{+\infty} \vartheta^{(n)}(0) (\frac{1}{\sum_{j=1}^{N} a_{ij} \tilde{I}_j})^n}, \tag{21}$$

or

$$\frac{1}{\tilde{I}_i} = \frac{1}{\tilde{S}_i} \sum_{n=1}^{+\infty} \delta_{\text{eff}} \vartheta^{(n)}(0) (\frac{1}{\sum_{j=1}^{N} a_{ij} \tilde{I}_j})^n. \tag{22}$$

The higher order derivatives of the inverse function $\vartheta(\tau)$ can be calculated as [1]

$$\vartheta^{(1)}(0) = \frac{1}{\Omega^{(1)}(0)}. \tag{23}$$

For $n \geq 2$, we have

$$\vartheta^{(n)}(0) = \lim_{\Delta x \to 0} n! \frac{\Delta x - \sum_{i=1}^{n-1} \frac{1}{i!} (\vartheta^{(i)}(0)) [\sum_{j=1}^{n} \frac{1}{j!} (\Omega^{(j)}(0)) (\Delta x)^j]^i}{[\sum_{i=1}^{n} \frac{1}{i!} (\vartheta^{(i)}(0)) (\Delta x)^i]^n}. \tag{24}$$

For the non-Markovian dynamics to be equivalent to Markovian, it is necessary that Supplementary Eq. (22) be reduced to the form of the steady state equation for Markovian spreading dynamics. The necessary condition is then that the quantity $\vartheta^{(n)}(0)$ must be zero for $\forall n > 1$. In this case, Supplementary Eq. (22) can be rewritten as

$$\frac{1}{\tilde{I}_i} = \frac{\delta_{\text{eff}} \vartheta^{(1)}(0)}{\tilde{S}_i \sum_{j=1}^{N} a_{ij} \tilde{I}_j}, \tag{25}$$

Since $\vartheta^{(1)}(0)$ is a finite value, we have $\vartheta(\tau) = \vartheta^{(1)}(0)\tau$. The inverse function of $\vartheta(\tau)$ is $\Omega(\tau) = \tau / \vartheta^{(1)}(0)$. From Supplementary Eq. (15), we get the first-order and second-order derivatives of $\Omega(\tau)$ as

$$\Omega'(\tau) = \delta_{\text{eff}} \int_0^{\tau} \omega_{\text{inf}}(\tau') \Psi_{\text{rec}}(\tau') d\tau' + \delta_{\text{eff}} \int_{\tau}^{+\infty} \omega_{\text{inf}}(\tau) \Psi_{\text{rec}}(\tau') d\tau' = \frac{1}{\vartheta^{(1)}(0)} \tag{26}$$

and

$$\Omega''(\tau) = \delta_{\text{eff}} \omega'_{\text{inf}}(\tau) \int_{\tau}^{+\infty} \Psi_{\text{rec}}(\tau') d\tau' = 0. \tag{27}$$

Because $\int_{\tau}^{+\infty} \Psi_{\text{rec}}(\tau') d\tau'$ is a monotonically decreasing function of τ in Supplementary Eq. (27), we have $\omega'_{\text{inf}}(\tau) = 0$ and $\omega_{\text{inf}}(\tau) = 1/\vartheta^{(1)}(0)$ while the integral $\int_{\tau}^{+\infty} \Psi_{\text{rec}}(\tau') d\tau'$ does not decrease to zero. Using the fact that the infection time distribution follows the distribution

$$\psi_{\text{inf}}(\kappa) = \omega_{\text{inf}}(\kappa) e^{-\int_0^{\kappa} \omega_{\text{inf}}(\kappa') d\kappa'}, \tag{28}$$

we have

$$\psi_{\text{inf}}(\tau) = \frac{1}{\vartheta^{(1)}(0)} e^{\frac{-\tau}{\vartheta^{(1)}(0)}}, \tag{29}$$

which is an exponential time distribution typical of a Markovian process and strictly follows the stationary state of Supplementary Eq. (25). If, for $\tau = \tau_0$, the following integral vanishes,

$$\int_{\tau_0}^{+\infty} \Psi_{\text{rec}}(\tau') d\tau' = 0, \tag{30}$$

we then have $\omega'_{\rm inf}(\tau_0) \neq 0$ and $\omega_{\rm inf}(\tau_0) \neq 1/\vartheta^{(1)}(0)$. However, in this case the quantities $\omega'_{\rm inf}(\tau_0)$ and $\omega_{\rm inf}(\tau_0)$ are not physically meaningful because an infected node has recovered when the value of the integral $\int_{\tau}^{+\infty} \Psi_{\rm rec}(\tau')d\tau'$ decreases to zero. Taken together, the analysis enables us to draw the conclusion that, for SIS processes with type-I activation mechanism, only when the infection times are distributed exponentially will the whole process be equivalent to a Markovian process.

SUPPLEMENTARY NOTE 2. THEORETICAL ANALYSIS OF NON-MARKOVIAN SIS MODEL WITH TYPE-II ACTIVATION MECHANISM

We first derive the infection rate $\eta(\tau)$ of infected node j of age τ. Define $A_{l\leftarrow i}(\kappa,\tau;t)$ as the two-dimensional probability density function that the edge $l \leftarrow i$ is active with age κ and the infection age of node i is τ. The probability density function that node i stays in the infected state aged τ at time t is given by

$$I_i(\tau;t) = \lim_{\Delta\kappa\to 0} \sum_{n=0}^{\frac{\tau}{\Delta\kappa}} A_{l\leftarrow i}(n\Delta\kappa,\tau;t)\Delta\kappa = \int_0^{\tau} A_{l\leftarrow i}(\kappa,\tau;t)d\kappa, \tag{31}$$

It is worth noting that, for $\tau = 0$, the following equality holds:

$$I_i(0;t) = \lim_{\Delta\kappa\to 0} A_{l\leftarrow i}(0,0;t)\Delta\kappa = A_{l\leftarrow i}(0,0;t)d\kappa. \tag{32}$$

At time t, the probability that the edge $l \leftarrow i$ is active with age ranging from κ to $\kappa+d\kappa$ and node i is in the infected state with infection age in the interval $(\tau,\tau+d\tau)$ is $A_{l\leftarrow i}(\kappa,\tau,t)d\kappa d\tau$. In this case, node i returns to the susceptible state with probability $\omega_{\rm rec}(\tau)d\tau$. The evolution equation of $I_i(\tau;t)$ is

$$(\frac{\partial}{\partial\tau} + \frac{\partial}{\partial t})I_i(\tau;t) = -\omega_{\rm rec}(\tau)I_i(\tau;t). \tag{33}$$

The probability that the edge $l \leftarrow i$ transmits disease outward is $\omega_{\rm inf}(\kappa)d\kappa$. The probability that neither recovery nor transmission occurs is $1 - \omega_{\rm rec}(\tau)d\tau - \omega_{\rm inf}(\kappa)d\kappa$. The evolution equation of $A_{l\leftarrow i}(\kappa,\tau;t)$ can then be written as

$$(\frac{\partial}{\partial\kappa} + \frac{\partial}{\partial\tau} + \frac{\partial}{\partial t})A_{l\leftarrow i}(\kappa,\tau;t) = -[\omega_{\rm inf}(\kappa) + \omega_{\rm rec}(\tau)]A_{l\leftarrow i}(\kappa,\tau;t). \tag{34}$$

Since an infected node i with infection age in the range from zero to $+\infty$ can transition into the susceptible state aged zero insofar as recovery is possible, we obtain the probability density function that node i returns to the susceptible state of zero age as

$$S_i(0;t+dt) = \int_0^{+\infty}\int_0^{\tau} \omega_{\rm rec}(\tau)A_{l\leftarrow i}(\kappa,\tau;t)d\kappa d\tau. \tag{35}$$

To describe the time evolution of $S_i(\tau;t)$, we assume that the ages of two connected nodes are uncorrelated, and thus $\Phi_{i\leftarrow j}(\tau;t)$ is the probability density function that node i in the susceptible state of age τ is infected by node j at time t. During the period $(t, t+dt)$, a susceptible node i aged τ is infected by its neighbors with probability $\sum_{j=1}^{N} a_{ij}\Phi_{i\leftarrow j}(\tau;t)d\tau$,

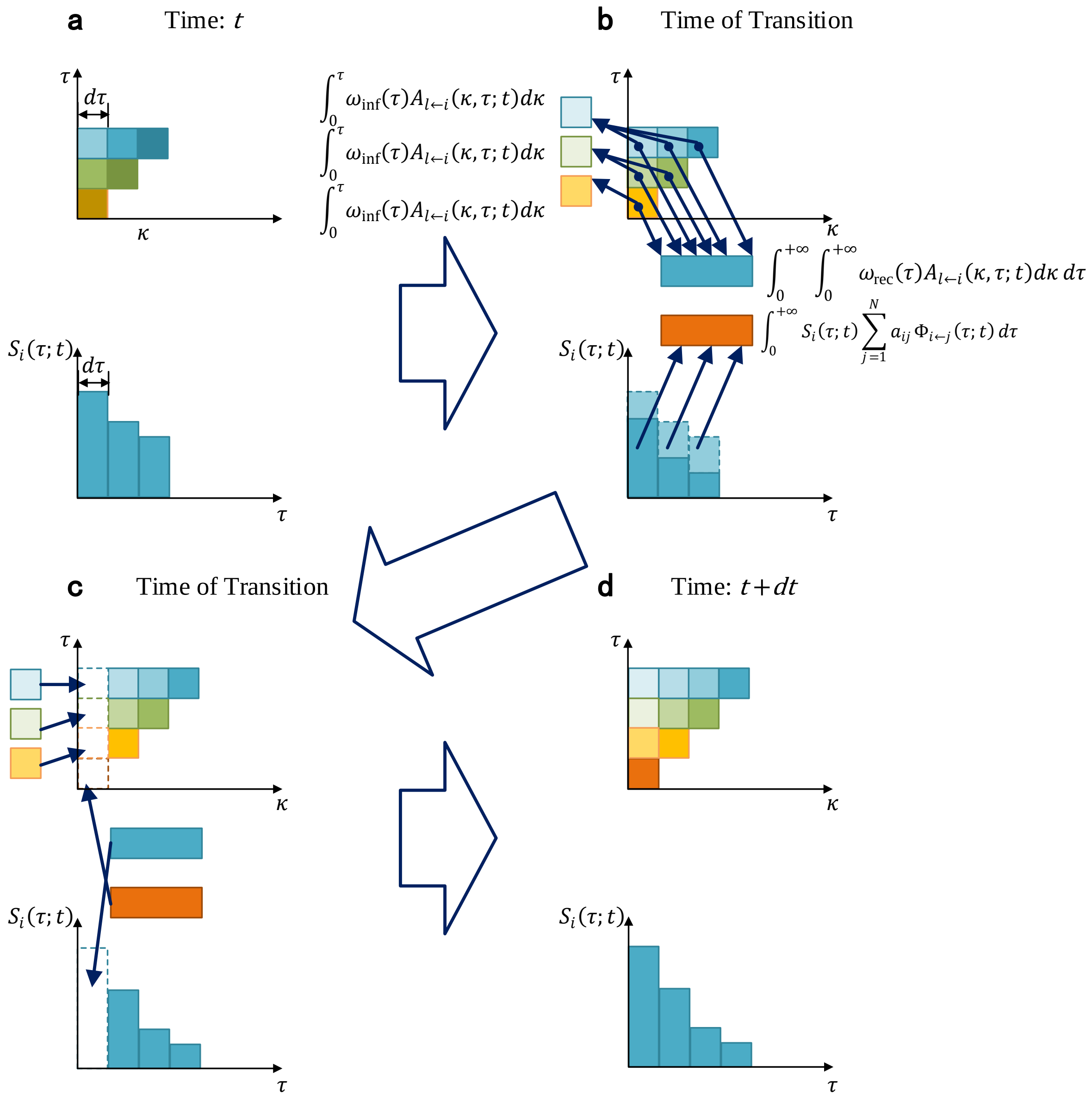

Supplementary Figure 1. Mechanism of probability flows with type-II activation mechanism. The magnitude of $A_{l\leftarrow i}(\kappa,\tau;t)$ is represented by the color of the squares, where a darker color indicates a larger value. **a** At time t, there are two distributions, $A_{l\leftarrow i}(\kappa,\tau;t)$ and $S_i(\tau;t)$. **b** Each square in $A_{l\leftarrow i}(\kappa,\tau;t)$ loses probability densities $\omega_{\text{inf}}(\kappa)d\kappa A_{l\leftarrow i}(\kappa,\tau;t)$ and $\omega_{\text{rec}}(\tau)d\tau A_{l\leftarrow i}(\kappa,\tau;t)$, and converges in the integrals $\int_0^{\tau}\omega_{\text{inf}}(\tau)A_{l\leftarrow i}(\kappa,\tau;t)d\kappa$ and $\int_0^{+\infty}\int_0^{\tau}\omega_{\text{rec}}(\tau)A_{l\leftarrow i}(\kappa,\tau;t)d\kappa d\tau$. Each block of $S_i(\tau;t)$ losses probability density $S_i(\tau,t)\sum_{j=1}^{N} a_{ij}\Phi_{i\leftarrow j}(\tau;t)d\tau$ and converges in the integral $\int_0^{+\infty} S_i(\tau,t)\sum_{j=1}^{N} a_{ij}\Phi_{i\leftarrow j}(\tau;t)d\tau$. **c** An amount of the density $\int_0^{+\infty}\int_0^{\tau}\omega_{\text{rec}}(\tau)A_{l\leftarrow i}(\kappa,\tau;t)d\kappa d\tau$ flows into the susceptible state, i.e., $S_i(0;t+dt)$, an amount $\int_0^{\tau}\omega_{\text{inf}}(\tau)A_{l\leftarrow i}(\kappa,\tau;t)d\kappa$ flows into the ordinate axis of $A_{l\leftarrow i}(\kappa,\tau;t+dt)$, i.e., $A_{l\leftarrow i}(0,\tau+d\tau;t+dt)$, $\int_0^{+\infty} S_i(\tau,t)\sum_{j=1}^{N} a_{ij}\Phi_{i\leftarrow j}(\tau;t)d\tau$ flows into the infected state: $A_{l\leftarrow i}(0,0;t+dt)$. **d** A series of probabilities flows results in two distributions $A_{l\leftarrow i}(\kappa,\tau;t=dt)$ and $S_i(\tau;t+dt)$ at time $t+dt$.

where N is the network size and a_{ij} is the element of the network adjacency matrix. We thus have

$$(\frac{\partial}{\partial \tau} + \frac{\partial}{\partial t})S_i(\tau; t) = -S_i(\tau; t) \sum_{j=1}^{N} a_{ij} \Phi_{i \leftarrow j}(\tau; t), \tag{36}$$

where

$$\Phi_{i \leftarrow j}(\tau; t) = \int_0^{\tau} \int_0^{\tau'} \omega_{\text{inf}}(\kappa) A_{i \leftarrow j}(\kappa, \tau'; t) d\kappa d\tau'. \tag{37}$$

Regardless of the value of the active age, from zero to ∞, of the edge $l \leftarrow i$ in the active state, if it propagates the disease, it is still in the active state but of age zero. We thus have

$$A_{l \leftarrow i}(0, \tau + d\tau; t + dt) = \int_0^{\tau} \omega_{\text{inf}}(\tau) A_{l \leftarrow i}(\kappa, \tau; t) d\kappa. \tag{38}$$

Since a susceptible node i with any susceptibility age ranging from zero to ∞ can transition into the infected state aged zero insofar as the infection process occurs, we obtain the probability density function that the edge $l \leftarrow i$ becomes the active state aged $\tau = \kappa = 0$ as

$$A_{l \leftarrow i}(0, 0; t + dt) = \int_0^{+\infty} S_i(\tau; t) \sum_{j=1}^{N} a_{ij} \Phi_{i \leftarrow j}(\tau; t) d\tau. \tag{39}$$

Supplementary Fig. 1 illustrates the probability flow between $A_{l \leftarrow i}(\kappa, \tau; t)$ and $S_i(\tau; t)$ and those in their respective interiors. To reduce the dimension of the evolution equations, we define

$$\eta(\tau; t) = \frac{\int_0^{\tau} \omega_{\text{inf}}(\kappa) A_{i \leftarrow j}(\kappa, \tau; t) d\kappa}{I_j(\tau; t)}, \tag{40}$$

which is the infection rate $\eta(\tau; t)$ of an infected node j aged τ at time t. Equation (37) can be rewritten as

$$\Phi_{i \leftarrow j}(\tau; t) = \int_0^{\tau} \eta(\tau'; t) I_j(\tau'; t) d\tau'. \tag{41}$$

From Supplementary Eq. (34), we get

$$A_{i \leftarrow j}(\kappa, \tau; t) = A_{i \leftarrow j}(0, \tau - \kappa; t - \kappa) e^{-\int_0^{\kappa} \omega_{\text{inf}} \tau' + \omega_{\text{rec}}(\tau - \kappa - \tau') d\tau'}. \tag{42}$$

Because of the relations $\Psi_{\text{rec}}(\tau) = e^{-\int_0^{\tau} \omega_{\text{rec}}(\tau') d\tau'}$ and $\Psi_{\text{inf}}(\kappa) = e^{-\int_0^{\kappa} \omega_{\text{inf}}(\kappa') d\kappa'}$, we can rewrite Supplementary Eq. (42) as

$$A_{i \leftarrow j}(\kappa, \tau; t) = A_{i \leftarrow j}(0, \tau - \kappa; t - \kappa) \Psi_{\text{inf}}(\kappa) \frac{\Psi_{\text{rec}}(\tau)}{\Psi_{\text{rec}}(\tau - \kappa)}. \tag{43}$$

Equation (38) can then be rewritten as

$$A_{i \leftarrow j}(0, \tau + d\tau; t + dt) = \int_0^{\tau} \omega_{\text{inf}}(\kappa) A_{i \leftarrow j}(0, \tau - \kappa; t - \kappa) \Psi_{\text{inf}}(\kappa) \frac{\Psi_{\text{rec}}(\tau)}{\Psi_{\text{rec}}(\tau - \kappa)} d\kappa. \tag{44}$$

Dividing both sides of the equation by $I_j(\tau; t) = I_j(0; t - \tau)\Psi_{\text{rec}}(\tau)$ [from Supplementary Eq. (33)] and combining it with Supplementary Eq. (38), we have

$$\eta(\tau; t) = \frac{A_{i \leftarrow j}(0, \tau + d\tau; t + dt)}{I_j(0; t - \tau)\Psi_{\text{rec}}(\tau)} = \int_0^{\tau} \frac{A_{i \leftarrow j}(0, \tau - \kappa; t - \kappa)\psi_{\text{inf}}(\kappa)}{I_j(0; t - \tau)\Psi_{\text{rec}}(\tau - \kappa)} d\kappa. \tag{45}$$

From Supplementary Eqs. (32) and (38) as well as the condition $\Psi_{\text{rec}}(0)=1$, we get

$$\begin{aligned}\eta(\tau;t) &= \int_0^{\tau-d\tau} \frac{A_{i\leftarrow j}(0,\tau-\kappa;t-\kappa)\psi_{\text{inf}}(\kappa)}{I_j(0;t-\tau)\Psi_{\text{rec}}(\tau-\kappa)}d\kappa+\psi_{\text{inf}}(\tau) \\ &= \int_{d\tau}^{\tau} \frac{A_{i\leftarrow j}(0,\tau+d\tau-\kappa;t+dt-\kappa)\psi_{\text{inf}}(\kappa-d\tau)}{I_j(0;t-\tau)\Psi_{\text{rec}}(\tau+d\tau-\kappa)}d\kappa+\psi_{\text{inf}}(\tau).\end{aligned} \tag{46}$$

Because of the relation

$$\int_0^{d\tau} \frac{A_{i\leftarrow j}(0,\tau+d\tau-\kappa;t+dt-\kappa)\psi_{\text{inf}}(\kappa-d\tau)}{I_j(0;t-\tau)\Psi_{\text{rec}}(\tau+d\tau-\kappa)}d\kappa \to 0, \tag{47}$$

Supplementary Eq. (46) can be written as

$$\eta(\tau;t) = \int_0^{\tau} \frac{A_{i\leftarrow j}(0,\tau+d\tau-\kappa;t+dt-\kappa)\psi_{\text{inf}}(\kappa-d\tau)}{I_j(0;t-\tau)\Psi_{\text{rec}}(\tau+d\tau-\kappa)}d\kappa+\psi_{\text{inf}}(\tau). \tag{48}$$

Moreover, using the relations $\psi_{\text{inf}}(\kappa-d\tau)\to\psi_{\text{inf}}(\kappa)$ and $\Psi_{\text{rec}}(\tau+d\tau-\kappa)\to\Psi_{\text{rec}}(\tau-\kappa)$, we can rewrite Supplementary Eq. (48) as

$$\eta(\tau;t) = \int_0^{\tau} \frac{A_{i\leftarrow j}(0,\tau+d\tau-\kappa;t+dt-\kappa)\psi_{\text{inf}}(\kappa)}{I_j(0;t-\tau)\Psi_{\text{rec}}(\tau-\kappa)}d\kappa+\psi_{\text{inf}}(\tau). \tag{49}$$

With the definition of $\eta(\tau;t)$ in Supplementary Eq. (45), we get

$$\begin{aligned}\eta(\tau;t) &= \int_0^{\tau} \eta(\tau-\kappa;t-\kappa)\psi_{\text{inf}}(\kappa)d\kappa+\psi_{\text{inf}}(\tau) \\ &= \int_0^{\tau} \eta(\kappa;t-\tau+\kappa)\psi_{\text{inf}}(\tau-\kappa)d\kappa+\psi_{\text{inf}}(\tau)\end{aligned} \tag{50}$$

For $\forall t\geq 0$, we have

$$\eta(0;t)=\psi_{\text{inf}}(0). \tag{51}$$

Solving the partial derivative of t for Supplementary Eq. (50), we get

$$\eta_t'(\tau;t) = \int_0^{\tau} \eta_t'(\kappa;t-\tau+\kappa)\psi_{\text{inf}}(\tau-\kappa)d\kappa. \tag{52}$$

The partial derivative of t for $\eta(\tau;t)$ at $\tau=0$ is thus zero:

$$\eta_t'(0;t)=0, \tag{53}$$

and $\eta_t'(0;t-\tau)=0$. From Supplementary Eq. (52), we obtain the solution series: $\eta_t'(d\tau;t-\tau+d\tau)=0$, $\eta_t'(2d\tau;t-\tau+2d\tau)=0,\ldots,\eta_t'(\tau;t)=0$. We thus have

$$\forall t>0, \forall\tau\leq t, \eta_t'(\tau;t)=0, \tag{54}$$

where $\eta(\tau;t)$ is independent of t. Letting $\eta(\tau)=\eta(\tau;t)$, we rewrite Supplementary Eq. (50) as

$$\eta(\tau) = \int_0^{\tau} \eta(\kappa)\psi_{\text{inf}}(\tau-\kappa)d\kappa+\psi_{\text{inf}}(\tau). \tag{55}$$

Performing Laplace transform on both sides of the equation, we obtain the following integral equation:

$$\eta(\tau) = \frac{1}{2\pi i}\int_{\sigma-i\infty}^{\sigma+i\infty} \frac{\hat{\psi}_{\text{inf}}(s)}{1-\hat{\psi}_{\text{inf}}(s)} e^{s\tau} ds, \tag{56}$$

where [2]

$$\hat{\psi}_{\text{inf}}(s) = \int_0^{+\infty} \psi_{\text{inf}}(\tau) e^{-s\tau} d\tau. \tag{57}$$

Finally, Supplementary Eqs. (34), (36), (38) and (39) can be reduced to:

$$(\frac{\partial}{\partial\tau} + \frac{\partial}{\partial t}) I_i(\tau;t) = -\omega_{\text{rec}}(\tau) I_i(\tau;t), \tag{58}$$

$$(\frac{\partial}{\partial\tau} + \frac{\partial}{\partial t}) S_i(\tau;t) = -S_i(\tau,t)\sum_{j=1}^{N} a_{ij}\Phi_{i\leftarrow j}(\tau;t), \tag{59}$$

$$I_i(0;t+dt) = \int_0^{+\infty} S_i(\tau;t)\sum_{j=1}^{N} a_{ij}\Phi_{i\leftarrow j}(\tau;t) d\tau, \tag{60}$$

$$S_i(0;t+dt) = \int_0^{+\infty} \omega_{\text{rec}}(\tau) I_i(\tau;t) d\tau, \tag{61}$$

where

$$\Phi_{i\leftarrow j}(\tau;t) = \int_0^{t} \eta(\tau') I_i(\tau',t) d\tau'. \tag{62}$$

When the system reaches a steady state, we obtain the differential equations of probability density functions of node i being in the susceptible and infected states as

$$\frac{d\tilde{I}_i(\tau)}{d\tau} = -\omega_{\text{rec}}(\tau)\tilde{I}_i(\tau), \tag{63}$$

$$\frac{d\tilde{S}_i(\tau)}{d\tau} = -\tilde{S}_i(\tau)\sum_{j=1}^{N} a_{ij}\int_0^{+\infty} \eta(\tau')\tilde{I}_j(\tau') d\tau', \tag{64}$$

and

$$\tilde{I}_i(0) = \tilde{S}_i(0). \tag{65}$$

Solving these equations, we obtain

$$\delta_{\text{eff}}\tilde{I}_i = \frac{\tilde{S}_i}{\int_0^{+\infty} e^{-\delta_{\text{eff}}\int_0^{\tau}\int_0^{+\infty}\eta(\tau')\Psi_{\text{rec}}(\tau')d\tau' d\tau'' \sum_{j=1}^{N} a_{ij}\tilde{I}_j}}. \tag{66}$$

Defining

$$\lambda_{\text{eff}} \equiv \int_0^{+\infty} \eta(\tau)\Psi_{\text{rec}}(\tau) d\tau, \tag{67}$$

we can reduce the equation to

$$\tilde{I}_i = \lambda_{\text{eff}}\tilde{S}_i\sum_{j=1}^{N} a_{ij}\tilde{I}_j, \tag{68}$$

Combining Supplementary Eq. (56) and the definition of λ_{eff}:

$$\lambda_{\text{eff}} \equiv \int_0^{+\infty} \Psi_{\text{rec}}(\tau) \frac{1}{2\pi i} \int_{\sigma-i\infty}^{\sigma+i\infty} \frac{\hat{\psi}_{\text{inf}}(s)}{1-\hat{\psi}_{\text{inf}}(s)} e^{s\tau} d\tau ds, \tag{69}$$

and exchanging the integration order, we obtain

$$\lambda_{\text{eff}} = \frac{1}{2\pi i} \int_{\sigma-i\infty}^{\sigma+i\infty} \frac{\hat{\psi}_{\text{inf}}(s)[\hat{\psi}_{\text{rec}}(-s)-1]}{1-\hat{\psi}_{\text{inf}}(s)} \frac{ds}{s}, \tag{70}$$

where

$$\hat{\psi}_{\text{rec}}(s) = \int_0^{+\infty} \psi_{\text{rec}}(\tau) e^{-s\tau} d\tau. \tag{71}$$

Note that

$$\frac{1}{2\pi i} \int_{\sigma-i\infty}^{\sigma+i\infty} \frac{\hat{\psi}_{\text{inf}}(s)}{1-\hat{\psi}_{\text{inf}}(s)} \frac{ds}{s} = 0, \tag{72}$$

when we take the integration path as a contour of an infinite radius, i.e., $s = re^{i\theta}$, where $r \to +\infty$, $-\frac{\pi}{2} < \theta < \frac{\pi}{2}$, and

$$\lim_{r\to+\infty} \frac{\hat{\psi}_{\text{inf}}(re^{i\theta})}{1-\hat{\psi}_{\text{inf}}(re^{i\theta})} = 0. \tag{73}$$

We thus have

$$\lambda_{\text{eff}} = \frac{1}{2\pi i} \int_C \frac{\hat{\psi}_{\text{inf}}(s)\hat{\psi}_{\text{rec}}(-s)}{1-\hat{\psi}_{\text{inf}}(s)} \frac{ds}{s}, \tag{74}$$

where C is a contour that encloses the entire $Re(s) > 0$ region [2].

SUPPLEMENTARY NOTE 3. SECOND-ORDER MEAN FIELD THEORY AND ERROR ANALYSIS

A. Second-order mean field theory

For SIS type of spreading dynamics, there are three distinct types of mean field theories, in the order of increase in the prediction accuracy and computational complexity: (1) the classical mean field theory based on the assumption that all nodes are equivalent [3], (2) heterogeneous mean field theory in which all the nodes with the same degree are assumed to be equivalent to each other [4], and (3) quench mean field theory where the state changes of individual nodes are treated separately [5, 6]. In these theories, the dynamical correlation between any pair of connected nodes is ignored, i.e., the states of any two connected nodes are regarded as independent. The three types of theories thus belong to first-order mean field analysis. To capture the dynamical correlation, a second-order approximation taking into account the evolution of the states of nodal pairs together is needed [7–13]. In fact, the pairwise approximation approach is a second-order theory, which regards nodal pairs with the same degree as equivalent [7–9, 13]. The master equation approach deals with not only the state changes of the nodes with the same degree, but also the state changes of their

neighbors. With approximations, it can be reduced to the evolution equations of pairwise approximation and mean field framework [14].

Here we develop a second-order mean field theory to deal with non-Markovian SIS dynamics. We first define the states of every pair of nodes. Specifically, for each pair of nodes, there are four different states: the $\langle II\rangle$ or $\langle AA\rangle$ state indicating that both nodes have been infected, the $\langle SI\rangle$ or $\langle SA\rangle$ state denoting that one node is susceptible and the other has been infected, the $\langle IS\rangle$ or $\langle AS\rangle$ state specifying that one node has been infected and the other is susceptible, and the $\langle SS\rangle$ state signifying that both nodes are susceptible. Note that the nodal pair states $\langle II\rangle$, $\langle SI\rangle$, $\langle IS\rangle$ are for type-I edge activation mechanism, and $\langle AA\rangle$, $\langle SA\rangle$, $\langle AS\rangle$ are for type-II edge activation mechanism.

The computational complexity required by the second-order mean field theory can often be prohibitively high. To make an analysis feasible, we restrict our study to the homogeneous case where the nodal pairs are equivalent in the Erdös-Rényi (ER) type of random networks. For type-I edge activation mechanism, we define $\langle II\rangle(\tau_1,\tau_2;t)$ as the probability density function that one node in a pair is in the infected state with the age of τ_1 and the other node is in the susceptible state with the age of τ_2 at time t. Similarly, the probabilities $\langle SI\rangle(\tau_1,\tau_2;t)$, $\langle IS\rangle(\tau_1,\tau_2;t)$, and $\langle SS\rangle(\tau_1,\tau_2;t)$ can be defined. The evolution equations of the pair node states for type-I activation are

$$(\frac{\partial}{\partial\tau}+\frac{\partial}{\partial t})I(\tau;t)=-\omega_{\mathrm{rec}}(\tau)I(\tau;t), \tag{75}$$

$$S(0;t+dt)=\int_0^{+\infty}\omega_{\mathrm{rec}}(\tau)I(\tau;t)d\tau, \tag{76}$$

$$(\frac{\partial}{\partial\tau}+\frac{\partial}{\partial t})S(\tau;t)=-S(\tau;t)\langle k\rangle\Phi(\tau;t), \tag{77}$$

$$I(0;t+dt)=\int_0^{+\infty}S(\tau;t)\langle k\rangle\Phi(\tau;t)d\tau, \tag{78}$$

$$(\frac{\partial}{\partial\tau_1}+\frac{\partial}{\partial\tau_2}+\frac{\partial}{\partial t})\langle II\rangle(\tau_1,\tau_2;t)=-[\omega_{\mathrm{rec}}(\tau_1)+\omega_{\mathrm{rec}}(\tau_2)]\langle II\rangle(\tau_1,\tau_2;t), \tag{79}$$

$$\langle SI\rangle(0,\tau_2;t)=\int_0^{+\infty}\omega_{\mathrm{rec}}(\tau_1)\langle II\rangle(\tau_1,\tau_2;t)d\tau_1, \tag{80}$$

$$\langle IS\rangle(\tau_1,0;t)=\int_0^{+\infty}\omega_{\mathrm{rec}}(\tau_2)\langle II\rangle(\tau_1,\tau_2;t)d\tau_2, \tag{81}$$

$$\begin{aligned}&(\frac{\partial}{\partial\tau_1}+\frac{\partial}{\partial\tau_2}+\frac{\partial}{\partial t})\langle SI\rangle(\tau_1,\tau_2;t)\\ =&-\{(\langle k\rangle-1)\Phi(\tau_1;t)+\omega_{\mathrm{inf}}[min(\tau_1,\tau_2)]+\omega_{\mathrm{rec}}(\tau_2)\}\langle SI\rangle(\tau_2,\tau_2;t),\end{aligned} \tag{82}$$

$$\langle II\rangle(0,\tau_2;t)=\int_0^{+\infty}\{(\langle k\rangle-1)\Phi(\tau_1;t)+\omega_{\mathrm{inf}}[min(\tau_1,\tau_2)]\}\langle SI\rangle(\tau_1,\tau_2;t)d\tau_1, \tag{83}$$

$$\langle SS\rangle(\tau_1,0;t)=\int_0^{+\infty}\omega_{\mathrm{rec}}(\tau_2)\langle SI\rangle(\tau_1,\tau_2;t)d\tau_2, \tag{84}$$

$$\begin{aligned}&(\frac{\partial}{\partial\tau_1}+\frac{\partial}{\partial\tau_2}+\frac{\partial}{\partial t})\langle IS\rangle(\tau_1,\tau_2;t)\\ =&-\{\omega_{\mathrm{rec}}(\tau_1)+(\langle k\rangle-1)\Phi(\tau_2;t)+\omega_{\mathrm{inf}}[min(\tau_1,\tau_2)]\}\langle SI\rangle(\tau_1,\tau_2;t),\end{aligned} \tag{85}$$

$$\langle SS\rangle(0,\tau_2;t) = \int_0^{+\infty} \omega_{\text{rec}}(\tau_1)\langle IS\rangle(\tau_1,\tau_2;t)d\tau_1, \tag{86}$$

$$\langle II\rangle(\tau_1,0;t) = \int_0^{+\infty} \{(\langle k\rangle-1)\Phi(\tau_2;t)+\omega_{\text{inf}}[min(\tau_1,\tau_2)]\}\langle IS\rangle(\tau_1,\tau_2;t)d\tau_2, \tag{87}$$

$$\begin{aligned}&(\frac{\partial}{\partial\tau_1}+\frac{\partial}{\partial\tau_2}+\frac{\partial}{\partial t})\langle SS\rangle(\tau_1,\tau_2;t)\\ =&-[(\langle k\rangle-1)\Phi(\tau_1;t)+(\langle k\rangle-1)\Phi(\tau_2;t)]\langle SS\rangle(\tau_1,\tau_2;t),\end{aligned} \tag{88}$$

$$\langle IS\rangle(0,\tau_2;t) = \int_0^{+\infty} (\langle k\rangle-1)\Phi(\tau_1;t)\langle SS\rangle(\tau_1,\tau_2;t)d\tau_1, \tag{89}$$

$$\langle SI\rangle(\tau_1,0;t) = \int_0^{+\infty} (\langle k\rangle-1)\Phi(\tau_2;t)\langle SS\rangle(\tau_1,\tau_2;t)d\tau_2, \tag{90}$$

where

$$\Phi(\tau;t) = \int_0^{+\infty} \omega_{\text{inf}}[\min(\tau,\tau_2)]\frac{\langle SI\rangle(\tau_1,\tau_2;t)}{S(\tau;t)}d\tau_2. \tag{91}$$

Equations (79)-(81) describe that, in the $\langle II\rangle$ nodal pair state, recovery of the infected node with age τ_1 converts the pair into the $\langle SI\rangle$ state, and the second node recovers to turn the pair into the $\langle IS\rangle$ state. Equations (82)-(84) represent that, in the $\langle SI\rangle$ state, infection of the susceptible node turns the nodal pair into the $\langle II\rangle$ state, while recovery of the infected node changes the pair into the $\langle SS\rangle$ state. Equations (85)-(87) dictate that, in $\langle IS\rangle$ nodal pair state, recovery of infected node switches the pair into the $\langle SS\rangle$ state while infection of the susceptible node converts the pair into the $\langle II\rangle$ state. Equations (88)-(90) indicate that, in the $\langle SS\rangle$ state, infection of the susceptible node with age τ_1 transforms the pair into the $\langle SI\rangle$ state, while infection of the susceptible node with age τ_2 converts the pair into the $\langle IS\rangle$ state.

For type-II edge activation mechanism, we define $\langle AA\rangle(\kappa_1,\tau_1,\kappa_2,\tau_2;t)$ as the probability density function that a directed edge from an infected node with age κ_1 to an infected node with age κ_2 is in the active state at age κ_1, while the inverted edge is in the active state with age κ_2. We define the probability density functions $\langle SA\rangle(\tau_1,\kappa_2,\tau_2;t)$, $\langle AS\rangle(\kappa_1,\tau_1,\tau_2;t)$, and $\langle SS\rangle(\tau_1,\tau_2;t)$ accordingly. The evolution equations of a nodal pair states can be written as

$$(\frac{\partial}{\partial\kappa}+\frac{\partial}{\partial\tau}+\frac{\partial}{\partial t})A(\kappa,\tau;t) = -[\omega_{\text{inf}}(\kappa)+\omega_{\text{rec}}(\tau)]A(\kappa,\tau;t), \tag{92}$$

$$A(0,\tau+dt;t+dt) = \int_0^{+\infty} \omega_{\text{inf}}(\kappa)A(\kappa,\tau;t)d\kappa, \tag{93}$$

$$S(0;t+dt) = \int_0^{+\infty} \omega_{\text{rec}}(\tau)A(\kappa,\tau;t)d\tau, \tag{94}$$

$$(\frac{\partial}{\partial\tau}+\frac{\partial}{\partial t})S(\tau;t) = -S(\tau;t)\langle k\rangle\Phi(\tau;t), \tag{95}$$

$$A(0,0;t+dt) = \int_0^{+\infty} S(\tau;t)\langle k\rangle\Phi(\tau;t)d\tau, \tag{96}$$

$$\begin{aligned}&(\frac{\partial}{\partial\kappa_1}+\frac{\partial}{\partial\tau_1}+\frac{\partial}{\partial\kappa_2}+\frac{\partial}{\partial\tau_2}+\frac{\partial}{\partial t})\langle AA\rangle(\kappa_1,\tau_1,\kappa_2,\tau_2;t)\\ =&-[\omega_{\text{inf}}(\kappa_1)+\omega_{\text{rec}}(\tau_1)+\omega_{\text{inf}}(\kappa_2)+\omega_{\text{rec}}(\tau_2)]\langle AA\rangle(\kappa_1,\tau_1,\kappa_2,\tau_2;t),\end{aligned} \tag{97}$$

$$\langle AA\rangle(0,\tau_1,\kappa_2,\tau_2;t)=\int_0^{+\infty}\omega_{\text{inf}}(\kappa_1)\langle AA\rangle(\kappa_1,\tau_1,\kappa_2,\tau_2;t)d\kappa_1, \tag{98}$$

$$\langle SA\rangle(0,\kappa_2,\tau_2;t)=\int_0^{+\infty}\omega_{\text{rec}}(\tau_1)\langle AA\rangle(\kappa_1,\tau_1,\kappa_2,\tau_2;t)d\tau_1, \tag{99}$$

$$\langle AA\rangle(\kappa_1,\tau_1,0,\tau_2;t)=\int_0^{+\infty}\omega_{\text{inf}}(\kappa_2)\langle AA\rangle(\kappa_1,\tau_1,\kappa_2,\tau_2;t)d\kappa_2, \tag{100}$$

$$\langle AS\rangle(\kappa_1,\tau_1,0;t)=\int_0^{+\infty}\omega_{\text{rec}}(\tau_2)\langle AA\rangle(\kappa_1,\tau_1,\kappa_2,\tau_2;t)d\tau_2, \tag{101}$$

$$\begin{aligned}&(\frac{\partial}{\partial\tau_1}+\frac{\partial}{\partial\kappa_2}+\frac{\partial}{\partial\tau_2}+\frac{\partial}{\partial t})\langle SA\rangle(\tau_1,\kappa_2,\tau_2;t)\\=&-\{(\langle k\rangle-1)\Phi(\tau_1;t)+\omega_{\text{inf}}(\kappa_2)+\omega_{\text{rec}}(\tau_2)\}\langle SA\rangle(\tau_1,\kappa_2,\tau_2;t),\end{aligned} \tag{102}$$

$$\langle AA\rangle(0,0,\kappa_2,\tau_2;t)=\int_0^{+\infty}(\langle k\rangle-1)\Phi(\tau_1;t)\langle SA\rangle(\tau_1,\kappa_2,\tau_2;t)d\tau_1, \tag{103}$$

$$\langle AA\rangle(0,0,0,\tau_2;t)=\int_0^{+\infty}\omega_{\text{inf}}(\kappa_2)\langle SA\rangle(\tau_1,\kappa_2,\tau_2;t)d\kappa_2, \tag{104}$$

$$\langle SS\rangle(\tau_1,0;t)=\int_0^{+\infty}\int_0^{+\tau_2}\omega_{\text{rec}}(\tau_2)\langle SA\rangle(\tau_1,\kappa_2,\tau_2;t)d\kappa_2d\tau_2, \tag{105}$$

$$\begin{aligned}&(\frac{\partial}{\partial\kappa_1}+\frac{\partial}{\partial\tau_1}+\frac{\partial}{\partial\tau_2}+\frac{\partial}{\partial t})\langle AS\rangle(\kappa_1,\tau_1,\tau_2;t)\\=&-\{\omega_{\text{inf}}(\kappa_2)+\omega_{\text{rec}}(\tau_1)+(\langle k\rangle-1)\Phi(\tau_2;t)\}\langle AS\rangle(\kappa_1,\tau_1,\tau_2;t),\end{aligned} \tag{106}$$

$$\langle AA\rangle(0,\tau_1,0,0;t)=\int_0^{+\infty}\omega_{\text{inf}}(\kappa_1)\langle AS\rangle(\kappa_1,\tau_1,\tau_2;t)d\kappa_1, \tag{107}$$

$$\langle SS\rangle(0,\tau_2;t)=\int_0^{+\infty}\int_0^{+\tau_1}\omega_{\text{rec}}(\tau_1)\langle AS\rangle(\kappa_1,\tau_1,\tau_2;t)d\kappa_1d\tau_1, \tag{108}$$

$$\langle AA\rangle(\kappa_1,\tau_1,0,0;t)=\int_0^{+\infty}(\langle k\rangle-1)\Phi(\tau_2;t)\langle AS\rangle(\kappa_1,\tau_1,\tau_2;t)d\tau_2, \tag{109}$$

$$\begin{aligned}&(\frac{\partial}{\partial\tau_1}+\frac{\partial}{\partial\tau_2}+\frac{\partial}{\partial t})\langle SS\rangle(\tau_1,\tau_2;t)\\=&-[(\langle k\rangle-1)\Phi(\tau_1;t)+(\langle k\rangle-1)\Phi(\tau_2;t)]\langle SS\rangle(\tau_1,\tau_2;t),\end{aligned} \tag{110}$$

$$\langle AS\rangle(0,0,\tau_2;t)=\int_0^{+\infty}(\langle k\rangle-1)\Phi(\tau_1;t)\langle SS\rangle(\tau_1,\tau_2;t)\tau_1, \tag{111}$$

$$\langle SA\rangle(\tau_1,0,0;t)=\int_0^{+\infty}(\langle k\rangle-1)\Phi(\tau_2;t)\langle SS\rangle(\tau_1,\tau_2;t)\tau_2, \tag{112}$$

where

$$\Phi(\tau;t)=\int_0^{+\infty}\int_0^{+\tau_2}\omega_{\text{inf}}(\kappa_2)\frac{\langle SA\rangle(\tau_1,\kappa_2,\tau_2;t)}{S(\tau;t)}d\kappa_2d\tau_2. \tag{113}$$

Equations (98)-(101), respectively, describe that in the $\langle AA\rangle$ nodal pair state, disease transmission from an infected node with age τ_1 and directed edge age κ_1 to an infected node of age τ_2 and directed edge age κ_2 converts the nodal pair into the $\langle AA\rangle$ state with $\kappa_1=0$, the recovery of an infected node with age τ_1 transforms the pair into the $\langle SA\rangle$ state, disease transmission from the infected node with age τ_2 and directed edge age κ_2 to the infected

node of age τ_1 and directed edge age κ_1 turns the pair into the $\langle AA\rangle$ state with $\kappa_2 = 0$, and the recovery of the infected node with age τ_2 places the pair into the $\langle AS\rangle$ state. Similarly, Supplementary Eqs. (102)-(105), respectively, indicate that in the $\langle SA\rangle$ state, the susceptible node is infected by its neighbors other than the infected node aged at τ_2 so that the pair is turned into the $\langle AA\rangle$ state, the susceptible node is infected by the infected node of τ_2 and so the pair gets into the $\langle AA\rangle$ state, and recovery of the infected node aged at τ_2 places the pair into the $\langle SS\rangle$ state. Equations (106)-(109) indicate, respectively, that in the $\langle AS\rangle$ state, disease transmission from an infected node with age τ_1 and directed edge age κ_1 to a susceptible node of age τ_2 changes the pair into the $\langle AA\rangle$ state, recovery of the infected node with age τ_1 transforms the pair into the $\langle SS\rangle$ state, and the susceptible node is infected by its neighbors other than the infected node and thus the nodal pair transitions into the $\langle AA\rangle$ state. Equations (110), (111), and (112) mean, respectively, that for the $\langle SS\rangle$ state pair, the susceptible node aged at τ_1 is infected and thus the pair moves into the $\langle AS\rangle$ state, and the susceptible node aged at τ_2 is infected and so the pair changes into the $\langle SA\rangle$ state.

To verify the accuracy of the second-order mean field theory, we set the infection time and recovery time distributions to be the Weibullean type. Supplementary Fig. 2 shows that the theory is capable of predicting the simulation results more accurately than the first-order theory for ER random networks, even for the situation where the disease has a high decay rate [e.g., $\alpha_{\rm I} = 4$ in Supplementary Figs. 2(c) and 2(d)].

B. Error Analysis

Our development of the second-order theory, due to its relatively high accuracy, enables an analysis of the source of errors in the first-order mean field theory through a comparison between the results from the first-order and the second-order theories. (For a network with N nodes, the most accurate description is the Nth-order mean field theory, but it is practically infeasible to analyze theories with order higher than two.) Because the dynamical correlation between any nodal pair is completely ignored in the first-order theory, the error analysis enables an understanding of the effects of dynamical correlation on non-Markovian dynamics with respect to different edge activation mechanisms.

To begin a systematic error analysis, we rewrite the relevant equations from the first-order theory in a similar form to those in the second-order theory. Recall that the assumptions employed in the first-order theory, which are

$$\langle II\rangle(\tau_1,\tau_2;t) = I(\tau_1;t)I(\tau_2;t), \tag{114}$$
$$\langle SI\rangle(\tau_1,\tau_2;t) = S(\tau_1;t)I(\tau_2;t), \tag{115}$$
$$\langle IS\rangle(\tau_1,\tau_2;t) = I(\tau_1;t)S(\tau_2;t), \tag{116}$$
$$\langle SS\rangle(\tau_1,\tau_2;t) = S(\tau_1;t)S(\tau_2;t), \tag{117}$$
$$\langle AA\rangle(\kappa_1,\tau_1,\kappa_2,\tau_2;t) = A(\kappa_1,\tau_1;t)A(\kappa_2,\tau_2;t), \tag{118}$$
$$\langle SA\rangle(\tau_1,\kappa_2,\tau_2;t) = S(\tau_1;t)A(\kappa_2,\tau_2;t), \tag{119}$$
$$\langle AS\rangle(\kappa_1,\tau_1,\tau_2;t) = A(\kappa_1,\tau_1;t)S(\tau_2;t). \tag{120}$$

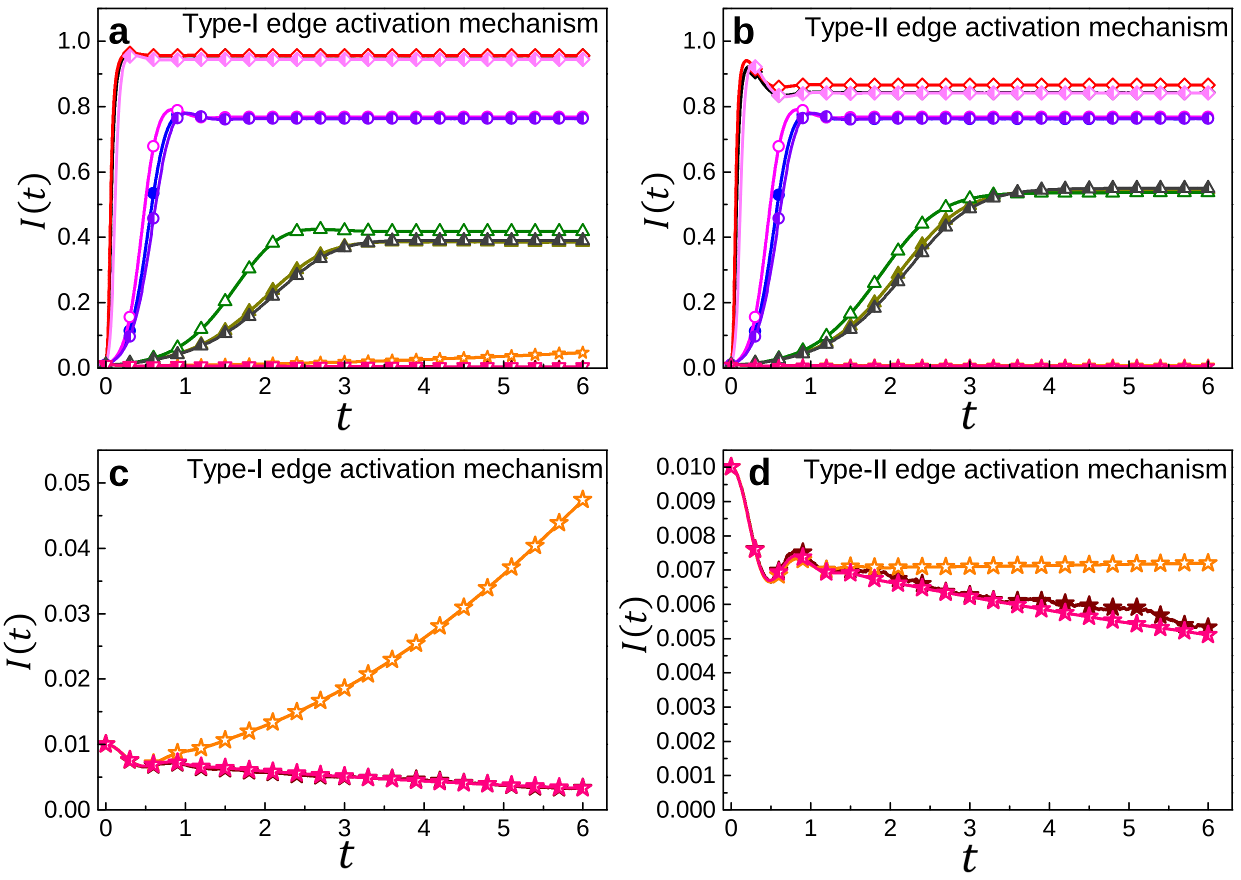

Supplementary Figure 2. Comparison of time evolution between simulated and theoretical results. **a**,**b** Time evolution for type-I and type-II mechanism, respectively. **c**,**d** Extinction process with $\alpha_{\rm I} = 4$ for type-I and type-II mechanism, respectively. In all panels, the solid symbols represent the simulation results averaged over 100 realizations on ER random networks, the open symbols represent the results of theoretical solutions obtained from the first-order mean field theory, and the half solid symbols correspond to the theoretical results from Supplementary Eqs. (75)-(113) in the second-order theory. Diamonds, circles, triangles, and stars are for $\alpha_{\rm I} = 0.5, 1, 2, 4$, respectively. The random networks have size $N = 10^4$ and mean degree $\langle k \rangle \approx 10$. Other parameters are $\beta_{\rm I} = 1$, $\alpha_{\rm R} = 2$, and $\beta_{\rm R} = 0.5$.

For $\langle SI \rangle(\tau_1, \tau_2; t)$, we can obtain its evolution equation as

$$\begin{aligned}
&(\frac{\partial}{\partial \tau_1} + \frac{\partial}{\partial \tau_2} + \frac{\partial}{\partial t})[S(\tau_1;t)I(\tau_2;t)] \\
=&\frac{S(\tau_1+dt;t+dt)I(\tau_2+dt;t+dt) - S(\tau_1;t)I(\tau_2;t)}{dt} \\
=&S(\tau_1+dt;t+dt)\frac{I(\tau_2+dt;t+dt) - I(\tau_2;t)}{dt} + I(\tau_2;t)\frac{S(\tau_1+dt;t+dt) - S(\tau_1;t)}{dt} \\
=&S(\tau_1;t)(\frac{\partial}{\partial \tau_1} + \frac{\partial}{\partial t})I(\tau_2;t) + I(\tau_2;t)(\frac{\partial}{\partial \tau_1} + \frac{\partial}{\partial t})S(\tau_1;t) \\
=&-[\Phi(\tau_1;t) + \omega_{\rm rec}(\tau_2)]S(\tau_1;t)I(\tau_2;t),
\end{aligned} \tag{121}$$

$$I(0;t)I(\tau_2;t) = I(\tau_2;t)\int_0^{+\infty}\langle k\rangle S(\tau_1)d\tau_1 = \int_0^{+\infty}\langle k\rangle S(\tau_1)I(\tau_2;t)d\tau_1, \tag{122}$$

$$S(\tau_1;t)S(0;t) = S(\tau_1;t)\int_0^{+\infty}\omega_{\mathrm{rec}}(\tau_2)I(\tau_2)d\tau_2 = \int_0^{+\infty}\omega_{\mathrm{rec}}(\tau_2)S(\tau_1;t)I(\tau_2)d\tau_2. \tag{123}$$

For type-I edge activation mechanism, we can get another form of the first-order mean field theory as

$$(\frac{\partial}{\partial\tau_1}+\frac{\partial}{\partial\tau_2}+\frac{\partial}{\partial t})\langle II\rangle(\tau_1,\tau_2;t) = -[\omega_{\mathrm{rec}}(\tau_1)+\omega_{\mathrm{rec}}(\tau_2)]\langle II\rangle(\tau_1,\tau_2;t), \tag{124}$$

$$\langle SI\rangle(0,\tau_2;t) = \int_0^{+\infty}\omega_{\mathrm{rec}}(\tau_1)\langle II\rangle(\tau_1,\tau_2;t)d\tau_1, \tag{125}$$

$$\langle IS\rangle(\tau_1,0;t) = \int_0^{+\infty}\omega_{\mathrm{rec}}(\tau_2)\langle II\rangle(\tau_1,\tau_2;t)d\tau_2, \tag{126}$$

$$\begin{aligned}&(\frac{\partial}{\partial\tau_1}+\frac{\partial}{\partial\tau_2}+\frac{\partial}{\partial t})\langle SI\rangle(\tau_1,\tau_2;t)\\ =&-[\langle k\rangle\Phi(\tau_1;t)+\omega_{\mathrm{rec}}(\tau_2)]\langle SI\rangle(\tau_2,\tau_2;t),\end{aligned} \tag{127}$$

$$\langle II\rangle(0,\tau_2;t) = \int_0^{+\infty}\langle k\rangle\Phi(\tau_1;t)\langle SI\rangle(\tau_1,\tau_2;t)\tau_1, \tag{128}$$

$$\langle SS\rangle(\tau_1,0;t) = \int_0^{+\infty}\omega_{\mathrm{rec}}(\tau_2)\langle SI\rangle(\tau_1,\tau_2;t)\tau_2, \tag{129}$$

$$\begin{aligned}&(\frac{\partial}{\partial\tau_1}+\frac{\partial}{\partial\tau_2}+\frac{\partial}{\partial t})\langle IS\rangle(\tau_1,\tau_2;t)\\ =&-[\omega_{\mathrm{rec}}(\tau_1)+\langle k\rangle\Phi(\tau_2;t)]\langle IS\rangle(\tau_1,\tau_2;t),\end{aligned} \tag{130}$$

$$\langle SS\rangle(0,\tau_2;t) = \int_0^{+\infty}\omega_{\mathrm{rec}}(\tau_1)\langle IS\rangle(\tau_1,\tau_2;t)\tau_1, \tag{131}$$

$$\langle II\rangle(\tau_1,0;t) = \int_0^{+\infty}\langle k\rangle\Phi(\tau_2;t)\langle IS\rangle(\tau_1,\tau_2;t)d\tau_2, \tag{132}$$

$$\begin{aligned}&(\frac{\partial}{\partial\tau_1}+\frac{\partial}{\partial\tau_2}+\frac{\partial}{\partial t})\langle SS\rangle(\tau_1,\tau_2;t)\\ =&-[\langle k\rangle\Phi(\tau_1;t)+\langle k\rangle\Phi(\tau_2;t)]\langle SS\rangle(\tau_1,\tau_2;t),\end{aligned} \tag{133}$$

$$\langle IS\rangle(0,\tau_2;t) = \int_0^{+\infty}\langle k\rangle\Phi(\tau_1;t)\langle SS\rangle(\tau_1,\tau_2;t)d\tau_1, \tag{134}$$

$$\langle SI\rangle(\tau_1,0;t) = \int_0^{+\infty}\langle k\rangle\Phi(\tau_2;t)\langle SS\rangle(\tau_1,\tau_2;t)d\tau_2, \tag{135}$$

where

$$\begin{aligned}\Phi(\tau;t) &= \int_0^{+\infty}\omega_{\mathrm{inf}}[\min(\tau,\tau_2)]\frac{\langle SI\rangle(\tau_1,\tau_2;t)}{S(\tau;t)}d\tau_2\\ &= \int_0^{+\infty}\omega_{\mathrm{inf}}[\min(\tau,\tau_2)]I(\tau_2;t)d\tau_2.\end{aligned} \tag{136}$$

For type-II edge activation mechanism, we have

$$\begin{aligned}&(\frac{\partial}{\partial\kappa_1}+\frac{\partial}{\partial\tau_1}+\frac{\partial}{\partial\kappa_2}+\frac{\partial}{\partial\tau_2}+\frac{\partial}{\partial t})\langle AA\rangle(\kappa_1,\tau_1,\kappa_2,\tau_2;t)\\=&-[\omega_{\text{inf}}(\kappa_1)+\omega_{\text{rec}}(\tau_1)+\omega_{\text{inf}}(\kappa_2)+\omega_{\text{rec}}(\tau_2)]\langle AA\rangle(\kappa_1,\tau_1,\kappa_2,\tau_2;t),\end{aligned}\tag{137}$$

$$\langle AA\rangle(0,\tau_1,\kappa_2,\tau_2;t)=\int_0^{+\infty}\omega_{\text{inf}}(\kappa_1)\langle AA\rangle(\kappa_1,\tau_1,\kappa_2,\tau_2;t)d\kappa_1,\tag{138}$$

$$\langle SA\rangle(0,\kappa_2,\tau_2;t)=\int_0^{+\infty}\omega_{\text{rec}}(\tau_1)\langle AA\rangle(\kappa_1,\tau_1,\kappa_2,\tau_2;t)d\tau_1,\tag{139}$$

$$\langle AA\rangle(\kappa_1,\tau_1,0,\tau_2;t)=\int_0^{+\infty}\omega_{\text{inf}}(\kappa_2)\langle AA\rangle(\kappa_1,\tau_1,\kappa_2,\tau_2;t)d\kappa_2,\tag{140}$$

$$\langle AS\rangle(\kappa_1,\tau_1,0;t)=\int_0^{+\infty}\omega_{\text{rec}}(\tau_2)\langle AA\rangle(\kappa_1,\tau_1,\kappa_2,\tau_2;t)d\tau_2,\tag{141}$$

$$\begin{aligned}&(\frac{\partial}{\partial\tau_1}+\frac{\partial}{\partial\kappa_2}+\frac{\partial}{\partial\tau_2}+\frac{\partial}{\partial t})\langle SA\rangle(\tau_1,\kappa_2,\tau_2;t)\\=&-\{\langle k\rangle\Phi(\tau_1;t)+\omega_{\text{inf}}(\kappa_2)+\omega_{\text{rec}}(\tau_2)\}\langle SA\rangle(\tau_1,\kappa_2,\tau_2;t),\end{aligned}\tag{142}$$

$$\langle AA\rangle(0,0,\kappa_2,\tau_2;t)=\int_0^{+\infty}\langle k\rangle\Phi(\tau_1;t)\langle SA\rangle(\tau_1,\kappa_2,\tau_2;t)d\tau_1,\tag{143}$$

$$\langle SA\rangle(\kappa_1,\tau_1,0,\tau_2;t)=\int_0^{+\infty}\omega_{\text{inf}}(\kappa_2)\langle SA\rangle(\tau_1,\kappa_2,\tau_2;t)d\kappa_2,\tag{144}$$

$$\langle SS\rangle(\tau_1,0;t)=\int_0^{+\infty}\int_0^{+\tau_2}\omega_{\text{rec}}(\tau_2)\langle SA\rangle(\tau_1,\kappa_2,\tau_2;t)d\kappa_2 d\tau_2,\tag{145}$$

$$\begin{aligned}&(\frac{\partial}{\partial\kappa_1}+\frac{\partial}{\partial\tau_1}+\frac{\partial}{\partial\tau_2}+\frac{\partial}{\partial t})\langle AS\rangle(\kappa_1,\tau_1,\tau_2;t)\\=&-\{\omega_{\text{inf}}(\kappa_2)+\omega_{\text{rec}}(\tau_1)+\langle k\rangle\Phi(\tau_2;t)\}\langle AS\rangle(\kappa_1,\tau_1,\tau_2;t),\end{aligned}\tag{146}$$

$$\langle AS\rangle(0,\tau_2,\kappa_2,\tau_2;t)=\int_0^{+\infty}\omega_{\text{inf}}(\kappa_1)\langle AS\rangle(\kappa_1,\tau_1,\tau_2;t)d\kappa_1,\tag{147}$$

$$\langle SS\rangle_(0,\tau_2;t)=\int_0^{+\infty}\int_0^{+\tau_1}\omega_{\text{rec}}(\tau_1)\langle AS\rangle(\kappa_1,\tau_1,\tau_2;t)d\kappa_1 d\tau_1,\tag{148}$$

$$\langle AA\rangle(\kappa_1,\tau_1,0,0;t)=\int_0^{+\infty}\langle k\rangle\Phi(\tau_2;t)\langle AS\rangle(\kappa_1,\tau_1,\tau_2;t)d\tau_2,\tag{149}$$

$$\begin{aligned}&(\frac{\partial}{\partial\tau_1}+\frac{\partial}{\partial\tau_2}+\frac{\partial}{\partial t})\langle SS\rangle(\tau_1,\tau_2;t)\\=&-[\langle k\rangle\Phi(\tau_1;t)+\langle k\rangle\Phi(\tau_2;t)]\langle SS\rangle(\tau_1,\tau_2;t),\end{aligned}\tag{150}$$

$$\langle AS\rangle(0,0,\tau_2;t)=\int_0^{+\infty}\langle k\rangle\Phi(\tau_1;t)\langle SS\rangle(\tau_1,\tau_2;t)\tau_1,\tag{151}$$

$$\langle SA\rangle(\tau_1,0,0;t)=\int_0^{+\infty}\langle k\rangle\Phi(\tau_2;t)\langle SS\rangle(\tau_1,\tau_2;t)\tau_2,\tag{152}$$

where

$$\Phi(\tau;t) = \int_0^{+\infty}\int_0^{+\tau_2} \omega_{\text{inf}}(\kappa_2)\frac{\langle SA\rangle(\tau_1,\kappa_2,\tau_2;t)}{S(\tau;t)}d\kappa_2 d\tau_2 \\ = \int_0^{+\infty}\int_0^{+\tau_2} \omega_{\text{inf}}(\kappa_2)A(\kappa_2,\tau_2;t)d\kappa_2 d\tau_2. \tag{153}$$

For type-I edge activation mechanism, we compare Supplementary Eqs. (79)-(81) with Supplementary Eqs. (124)-(126) to assess the dynamical correlation of the $\langle II\rangle$ state. We find that the two groups of equations are essentially identical, so there is no dynamical correlation between two infected nodes. Comparing Supplementary Eqs. (82) and (83), we see that, in Supplementary Eqs. (127) and (128) $\omega_{\text{inf}}[\min(\tau_1,\tau_2)]$ is replaced by $\Phi(\tau_1;t)$, indicating that the first-order mean field theory ignores any temporal correlation between an infected node and a susceptible node, enlarging the bias in describing the $\langle SI\rangle$ state. For the $\langle IS\rangle$ state, the method of analysis is the same as that for the $\langle SI\rangle$ state. For the $\langle SS\rangle$ state, we compare Supplementary Eqs. (88)-(90) with Supplementary Eqs. (133)-(135) and find that the quantity $\langle k\rangle - 1$ is replaced by $\langle k\rangle$. This means that the first-order mean field theory attempts to include the non-existent process of infection between two susceptible nodes, which is another source of error.

For type-II edge activation mechanism, we compare Supplementary Eqs. (97)-(101) with Supplementary Eqs. (137)-(141) to assess the dynamical correlation of the $\langle AA\rangle$ state. Note that the two groups of equations are identical, so there is no dynamical correlation between any pair of infected nodes. For the $\langle SA\rangle$ state, we compare Supplementary Eqs. (102)-(103) with Supplementary Eqs. (142)-(143) and identify the simple replacement of $\langle k\rangle - 1$ by $\langle k\rangle$. In addition, the quantity $\langle AA\rangle(0,0,0,\tau_2;t)$ in Supplementary Eq. (144) is replaced by $\langle SA\rangle(\kappa_1,\tau_1,0,\tau_2;t)$ in Supplementary Eq. (104). In the first-order equations, the infection of a susceptible node from any of its neighbors and the disease transmission through the active edge are considered as two independent processes. Specifically, the susceptible node would be infected by all its neighbors including the infected node [see Supplementary Eq. (143)], but disease transmission through such an active edge cannot result in the infection of the susceptible node [see Supplementary Eq. (144)]. This means that the first-order mean field theory ignores the causality between disease transmission through an active edge and infection of the susceptible node. For the $\langle SS\rangle$ state, we compare Supplementary Eqs. (110)-(112) with Supplementary Eqs. (150)-(152), and find that $\langle k\rangle - 1$ in the latter is replaced by $\langle k\rangle$ in the former, which means that the first-order mean field erroneously takes into account the non-existent infection process between two susceptible nodes.

The error analysis indicates that the two activation rules have distinct effects of dynamical correlation on active edges. For type-I edge activation mechanism, the errors in the first-order mean field theory mainly result from the temporal correlations on active edges, while for type-II edge activation mechanism, the errors are caused by the causality between disease transmission through the active edge and infection of the susceptible node.

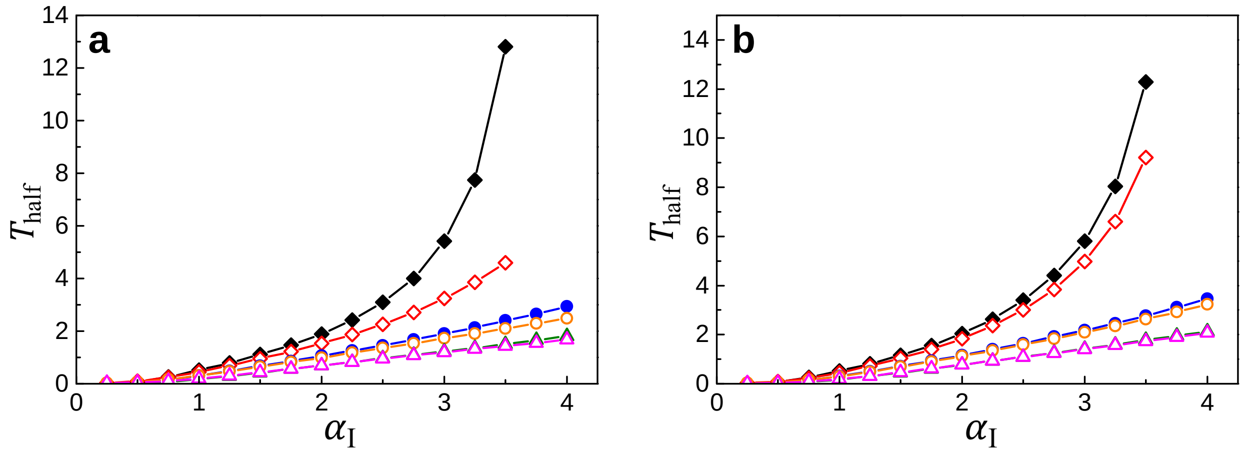

Supplementary Figure 3. Transient time versus $\alpha_{\rm I}$. The extent of the infection time distribution is characterized by $\alpha_{\rm I}$, where a smaller value of $\alpha_{\rm I}$ corresponds to a narrower distribution. Panels **a-b** are for results from the SIS model with type-I activation and type-II activation mechanisms, respectively. The solid and open symbols represent simulation and theoretical results, respectively. Diamonds, circles, and triangles, respectively, are for random, scale-free and Hamsterster networks.

SUPPLEMENTARY NOTE 4. EFFECT OF INFECTION TIME DISTRIBUTION ON TRANSIENT TIME

The effect of the inter-event time distribution on the speed of spreading dynamics is an issue of interest [15–19]. Because the time that the system has reached a steady state cannot be sharply defined, we set the empirical rule to determine the transient time T_{half} as the time at which the infected density reaches the average value between the initial density $I(0)$ and the steady-state density $\tilde{I}$. Supplementary Fig. 3 shows that a narrower distribution of the inter-event times, which corresponds to a smaller value of $\alpha_{\rm I}$, makes it easier for the system to reach a final steady state.

SUPPLEMENTARY NOTE 5. EFFECT OF DEGREE DISTRIBUTION ON TRANSIENT TIME

In general, the network topology can affect the spreading speed as well [20–25]. To address this issue, we investigate the relationship between transient lifetime and the degree distribution. To be concrete, we study the transient time on scale-free networks constructed from the uncorrelated configuration model [28]. Supplementary Fig. 4(a) shows that a smaller value of the power-law degree exponent γ enables the spreading dynamics to reach its steady state faster. The lower-cutoff degree $D_{\rm low}$ of the degree distribution can also affect the transient lifetime, as shown in Supplementary Fig. 4(b), where a larger value of $D_{\rm low}$ can also expedite the spreading dynamics in approaching the final steady state. These results indicate that effects of degree distribution on transient time for non-Markovian spreading dynamics are consistent with those for Markovian dynamics.

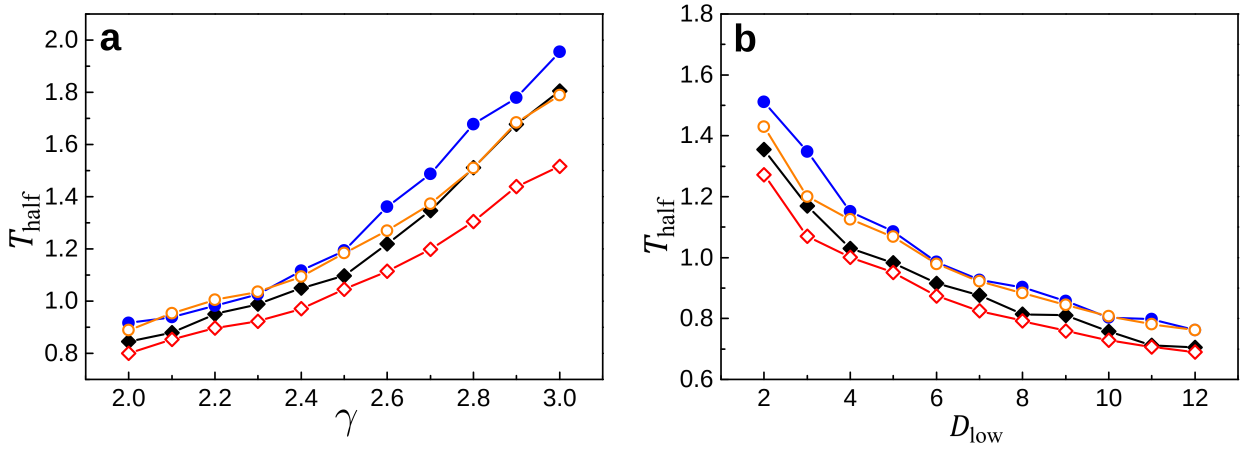

Supplementary Figure 4. Effect of network degree distribution on transient lifetime. Shown is the transient time versus two specific network structural parameter: power-law degree exponent γ and lower-cutoff degree D_{low}. In general, a smaller value of γ corresponds to the network with more hub nodes. **a-b** Results for SIS dynamics with respect to γ and D_{low}, respectively. The solid and open symbols represent simulation and theoretical results, respectively. Diamonds and circles are for type-I and type-II activation, respectively. In **a**, the value of D_{low} is fixed at five, while that of γ is set to be 2.3 in panel **b**. The networks have size $N = 10^4$ and the structural cutoff is characterized by the maximum degree $k_{max} \sim N^{1/2}$. Other parameters are $\alpha_{\text{I}} = 2$, $\beta_{\text{I}} = 1$, $\alpha_{\text{R}} = 2$, and $\beta_{\text{R}} = 0.5$.

SUPPLEMENTARY NOTE 6. STATIONARY PROBABILITY DENSITY DISTRIBUTIONS

To validate our proposed first-order mean field theory for non-Markovian processes, we examine the stationary probability density functions $\tilde{I}(\tau)$ and $\tilde{S}(\tau)$ defined as

$$\tilde{I}(\tau) = \frac{\sum_{i=1}^{N} \tilde{I}_i(\tau)}{N}, \tag{154}$$

$$\tilde{S}(\tau) = \frac{\sum_{i=1}^{N} \tilde{S}_i(\tau)}{N}, \tag{155}$$

and compare the theoretical predictions with the simulation results in Supplementary Fig. 5. Especially, we count the numbers of infected and susceptible nodes at every discrete state age (at the age difference $\Delta\tau = 0.001$) when the system reaches a steady state, and divide the numbers by $\Delta\tau \times N$ to get the simulated stationary probability density. The theoretical predictions agree with the simulation results for random, scale-free, and Hamsterster networks. The difference between SIS models with type-I and type-II edge activation mechanisms is that the results of $\tilde{S}(\tau)$ for the former decrease more smoothly than the latter in the region of small τ values for $\alpha_{\text{I}} = 2, 4$, indicating that newly recovered nodes in the latter are more susceptible to infection than the ones in the former. For $\alpha_{\text{I}} = 2, 4$, the infection rate increases with the age of the active edge. By type-II edge activation mechanism, the ages of the active edges depend only on the infected nodes and thus may be greater than those in type-I edge activation, resulting in some greater infection rate.

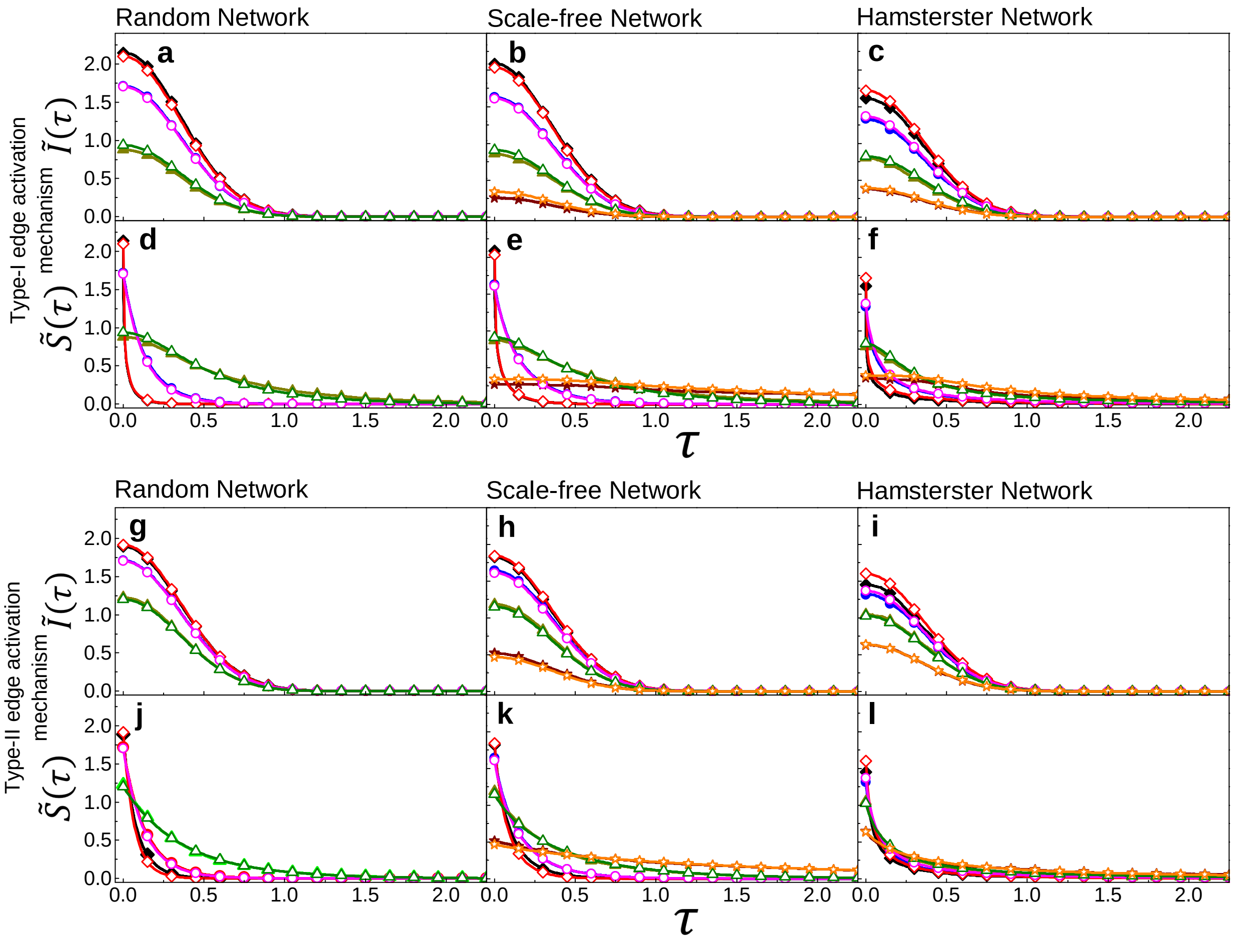

Supplementary Figure 5. Comparison of stationary probability density distributions between simulated and theoretical results. Panels in the first two rows show the results from the SIS model with type-I activation mechanism, and those in the last two rows present the results for the model with type-II mechanism. Panels in the first and third rows present the results of $\tilde{I}(\tau)$ while those in the second and fourth rows demonstrate the results of $\tilde{S}(\tau)$. Panels in the three columns (from left to right) are simulated or solved from theoretically derived evolution equations for random, scale-free and Hamsterster networks, respectively. The solid and open symbols represent simulation and theoretical results, respectively. Diamonds, circles, triangles, and stars, respectively, correspond to $\alpha_{\rm I} = 0.5, 1, 2, 4$. The random and scale-free networks have size $N = 10^4$ and mean degree $\langle k \rangle \approx 10$, and the Hamsterster network parameters are $N = 2426$ and $\langle k \rangle \approx 13.7$. Other parameters are $\beta_{\rm I} = 1$, $\alpha_{\rm R} = 2$, and $\beta_{\rm R} = 0.5$. Note that the lack of data points in the random network for $\alpha_{\rm I} = 4$ is due to the extinction of the infected density.

SUPPLEMENTARY NOTE 7. SPECIAL CASE I: MARKOVIAN EQUIVALENCE OF NON-MARKOVIAN SIS MODEL WITH TYPE-I EDGE ACTIVATION MECHANISM

For $\alpha_{\rm I} = 1$, the Weibull distribution reduces to an exponential distribution. In this case, the non-Markovian SIS with type-I activation mechanism is completely equivalent to

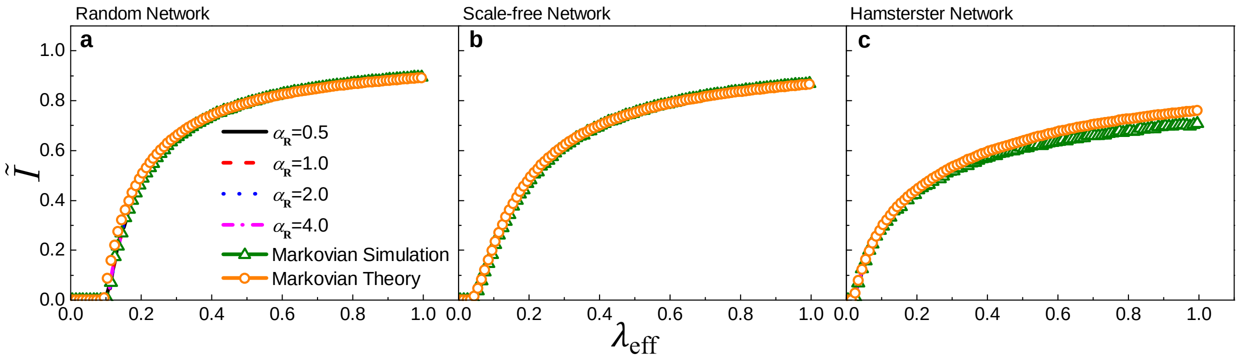

Supplementary Figure 6. Comparison of stationary infected density with type-I edge activation mechanism. Panels **a**-**c** are for results from random, scale-free, and Hamsterster networks, respectively. Solid, dashed, dotted, and dot-dashed curves, respectively, represent the results for $\alpha_{\rm R} = 0.5, 1, 2, 4$. Triangles and circles correspond to results from the Markovian process and the analytical solution, respectively. Other parameters are $\alpha_{\rm I} = 1$ and $\beta_{\rm R} = 0.5$.

a Markovian SIS process. We set

$$\psi_{\rm inf}(\kappa) = \frac{1}{\beta_{\rm I}} e^{-\frac{\kappa}{\beta_{\rm I}}}, \tag{156}$$

$$\psi_{\rm rec}(\tau) = \frac{\alpha_{\rm R}}{\beta_{\rm R}} (\frac{\tau}{\beta_{\rm R}})^{\alpha_{\rm R}-1} e^{-(\frac{\tau}{\beta_{\rm R}})^{\alpha_{\rm R}}}. \tag{157}$$

For $\alpha_{\rm R} = \{0.5, 1, 2, 4\}$ and $\beta_{\rm R} = 0.5$, we adjust the value of $\lambda_{\rm eff}$ through changing $\beta_{\rm I}$, where

$$\lambda_{\rm eff} = \frac{\beta_{\rm R}\Gamma(1/\alpha_{\rm R})}{\alpha_{\rm R}\beta_{\rm I}}, \tag{158}$$

and Γ is the gamma function. In Supplementary Figs. 6(a,b), we present results from Monte Carlo simulations for the three types of networks. The theoretical results from the Markovian process are obtained from a first-order mean field analysis. For all values of $\lambda_{\rm eff}$ (or $\beta_{\rm I}$), there is a good agreement between the infected density for $\alpha_{\rm I} = 1$ with the theoretical prediction of the Markovian model.

SUPPLEMENTARY NOTE 8. SPECIAL CASE II: NON-MARKOVIAN SPREADING ON STRONG HETEROGENEOUS NETWORKS

We test our theory on scale-free networks with a strong heterogeneous degree distribution, where the values of the network parameters are: size 10000, power-law degree exponent 2.3, lower cutoff degree being five, upper cutoff degree being about 100, and average degree approximately 11.9. A network in this ensemble has a large number of hub nodes. As shown in Supplementary Fig. 7, even in this relatively extreme case, our theory is able to predict the simulation results. Furthermore, when the spreading has reached the steady state, the approximate equivalence between non-Markovian and Markovian dynamics for both type-I and type-II activation mechanisms holds.

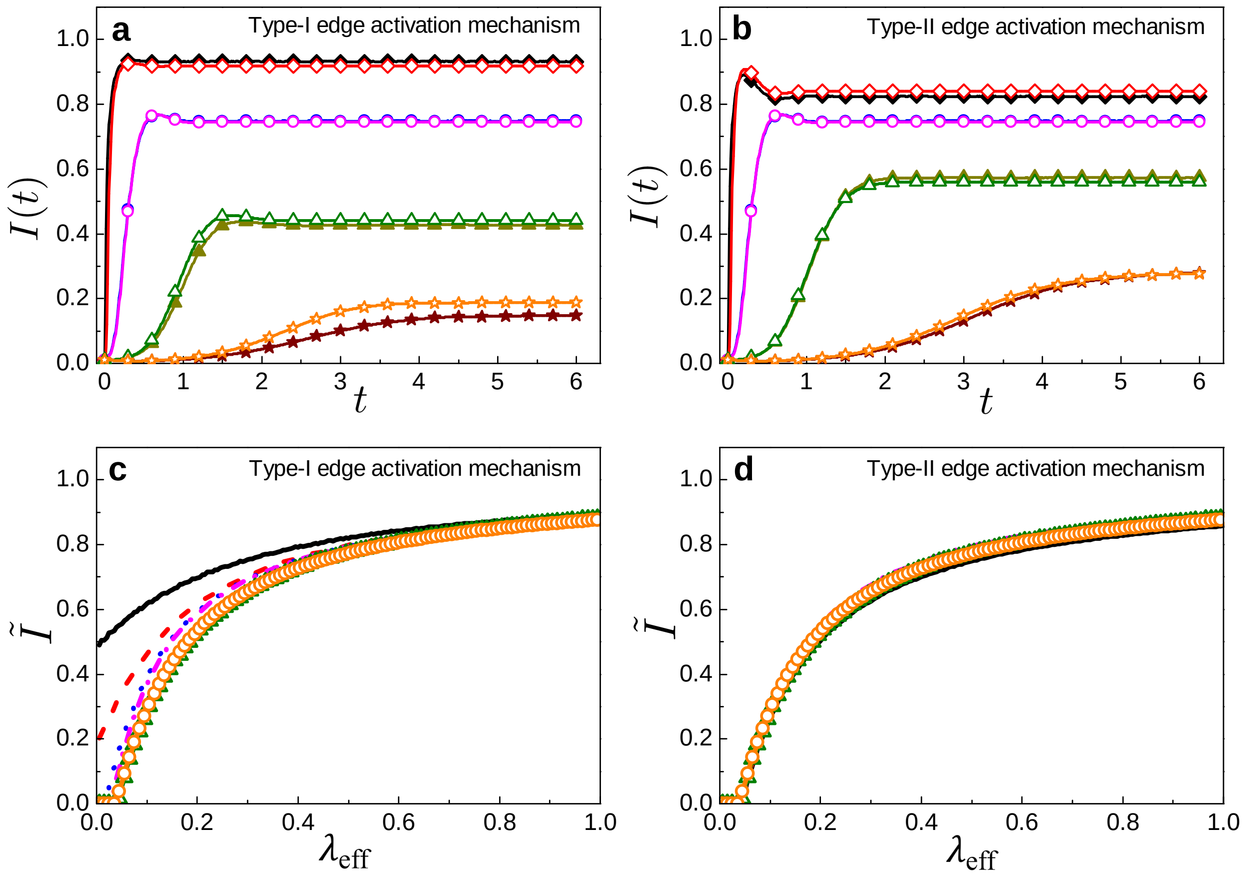

Supplementary Figure 7. Results on a scale-free network with strong heterogeneous degree distribution. **a**,**b** Comparison of time evolution between simulated and theoretical results for type-I and type-II activation mechanisms, respectively. The solid symbols represent the results of simulations which are averaged over 100 realizations. The open symbols denote the results of theoretical solutions. Diamonds, circles, triangles, and stars correspond to $\alpha_{\rm I} = 0.5, 1, 2, 4$, respectively. Other parameters are $\beta_{\rm I} = 1$, $\alpha_{\rm R} = 2$, and $\beta_{\rm R} = 0.5$. **c**,**d** Approximate equivalence between non-Markovian and Markovian processes with type-I and type-II activation mechanism, respectively. Solid, dashed, dotted, and dot-dashed curves, respectively, represent the results for $\alpha_{\rm R} = 0.5, 1, 2, 4$. Triangles and circles correspond to results from the Markovian process and analytical solutions, respectively. Other parameters are $\alpha_{\rm I} = 1$ and $\beta_{\rm R} = 0.5$.

SUPPLEMENTARY NOTE 9. SPECIAL CASE III: NON-MARKOVIAN SPREADING ON NETWORKS WITH TYPE-III EDGE ACTIVATION MECHANISM

Besides the two edge activation mechanisms in the main text, other types of edge activation mechanisms exist. For example, rule #2 in Ref. [26] prescribes that the age of an active link is solely determined by the age of the infected node [27], which is similar but not totally identical to type-II edge activation treated in the main text. For convenience, we call this rule as type-III edge activation mechanism.

Because of the absence of any temporal correlation on active edges in type-III activation, the evolution equations are similar to those for type-II edge activation [Supplementary

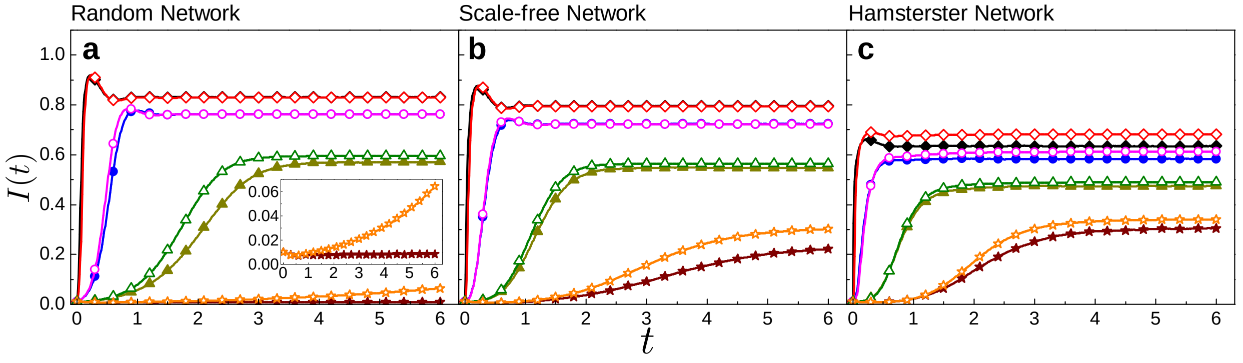

Supplementary Figure 8. Comparison of time evolution for type-III activation mechanism. The three panels from left to right are for random, scale-free, and Hamsterster networks, respectively. In all panels, the solid symbols represent the results of simulations which are averaged over 100 realizations for random and scale-free networks, and over 400 realizations for the Hamsterster network obtained from Eqs. (7-9) in the main text and Supplementary Eq. (159). The open symbols represent the results of theoretical solutions. Diamonds, circles, triangles, and stars correspond to $\alpha_{\rm I} = 0.5, 1, 2, 4$, respectively. The random and scale-free networks have size $N = 10^4$ and mean degree $\langle k \rangle \approx 10$, and the Hamsterster network has $N = 2426$ and $\langle k \rangle \approx 13.7$. The inset in panel **a** shows the extinction process with $\alpha_{\rm I} = 4$. Other parameters are $\beta_{\rm I} = 1$, $\alpha_{\rm R} = 2$, and $\beta_{\rm R} = 0.5$.

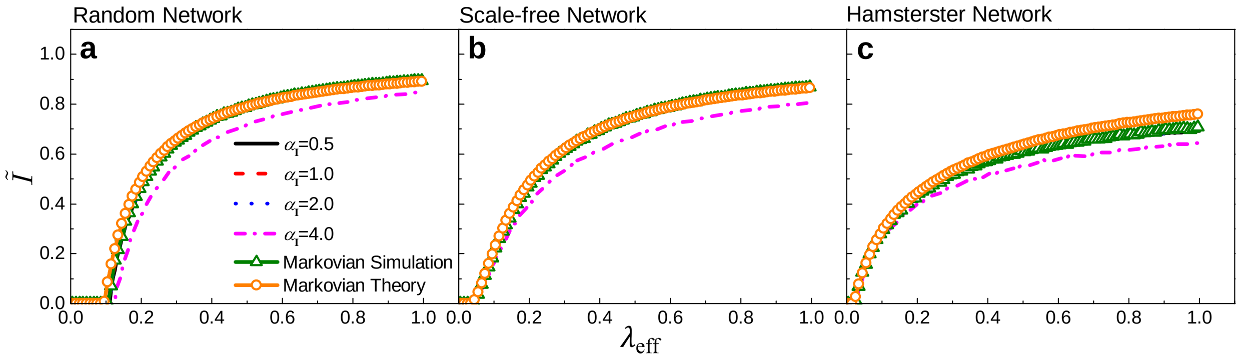

Supplementary Figure 9. Demonstration of equivalence with type-III edge activation mechanism. Comparison of stationary infected density between non-Markovian and Markovian processes. **a-c** Results from random, scale-free, and Hamsterster networks, respectively. Solid, dashed, dotted, and dot-dashed curves, respectively, represent the results for $\alpha_{\rm I} = 0.5, 1, 2, 4$. Triangles and circles represent simulation results from the Markovian process and the corresponding analytical solutions, respectively. Other parameters are $\alpha_{\rm R} = 2$ and $\beta_{\rm R} = 0.5$.

Eqs. (58) - (62)]. The only difference between these two activation mechanisms is the probability density function $\Phi_{i\leftarrow j}(\tau;t)$ in Supplementary Eq. (62). For type-III, $\Phi_{i\leftarrow j}(\tau;t)$ can be written as

$$\Phi_{i\leftarrow j}(\tau;t) = \int_0^t \omega_{\rm inf}(\tau') I_i(\tau',t) d\tau'. \tag{159}$$

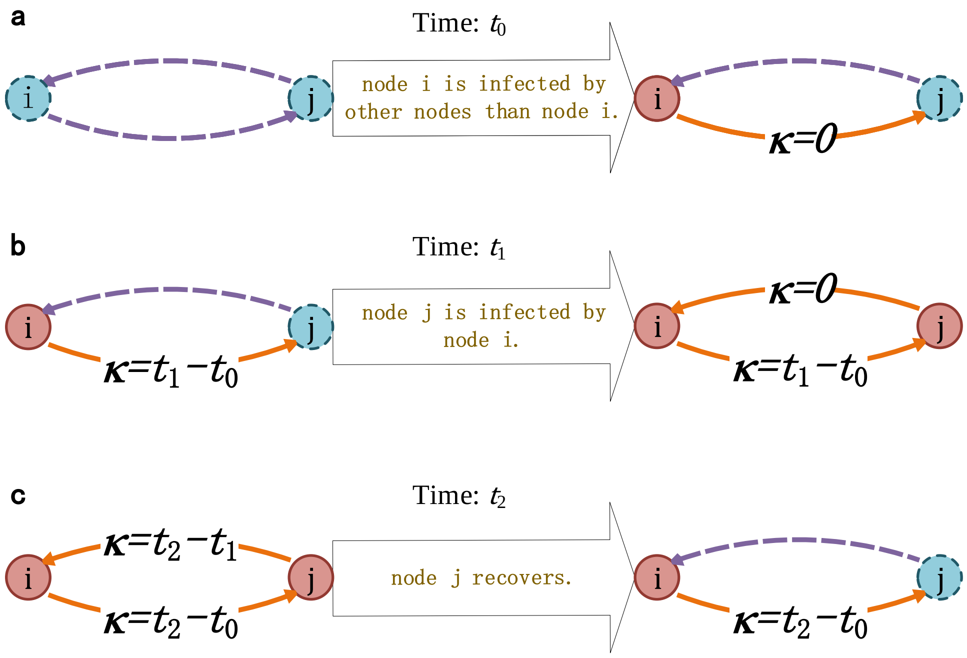

Supplementary Figure 10. An illustration of the growth of infection rate with type-III edge activation mechanism. The values of the relevant parameters are $\alpha_{\rm I} = 4.0$ and $\beta_{\rm I} = 1.0$, where the infection rate of an active age grows rapidly with time. **a** At $t_0 = 0$, node i is infected, and the active age is zero, so the infection rate is $\omega_{\rm inf}(0) = 0$. **b** At $t_1 = 1$, the active edge transmits disease and node j moves into the infected state, but the age of the active edge from node i to node j remains unchanged. In this case, the infection rate is $\omega_{\rm inf}(1) = 4$. **c** At time $t_2 = 2$, node j has recovered but node i is still in the infected state. The age of the active age is two. In this case, the infection rate is relatively large: $\omega_{\rm inf}(2) = 32$. As a result, node i will make node j infected again in a short time.

Equations (63)-(68) suggest an equivalence between SIS dynamics with type-II activation and Markovian SIS dynamics, the effective infection rate can be obtained as

$$\lambda_{\rm eff} \equiv \int_0^{+\infty} \eta(\tau)\Psi_{\rm rec}(\tau)d\tau. \tag{160}$$

We can then conclude that SIS dynamics with type-III activation is equivalent to Markovian SIS dynamics with the effective infection rate given by

$$\lambda_{\rm eff} \equiv \int_0^{+\infty} \omega_{\rm inf}(\tau)\Psi_{\rm rec}(\tau)d\tau. \tag{161}$$

To verify the above analysis, we simulate the dynamical processes and compare results with theoretical predictions. Supplementary Fig. 8 shows the time evolution of the infected density of the entire network for three different types of networks. We see that the mean-field predictions agree well with those of non-Markovian type of SIS spreading dynamics. We also find that a smaller value of $\alpha_{\rm I}$ results in a larger scale outbreak on the networks, which is consistent with the cases of type-I and type-II edge activation mechanisms.

We further test the equivalence between non-Markovian and Markovian spreading process with type-III activation mechanism. Representative results are shown in Supplementary Fig. 9. It can be seen that, regardless of the network structure, the stationary infected density for $\alpha_{\mathrm{I}} = 0.5, 1.0, 2.0$ agrees well with results from the simulation of Markovian process and from the analytical solution of the Markovian dynamics from Eq. (24) or Eq. (27) in the main text. However, the curve for $\alpha_{\mathrm{I}} = 4$ deviates markedly from the case of Markovian dynamics. To explain this phenomenon, we note that the infection rate is

$$\omega_{\mathrm{inf}}(\kappa) = \frac{\alpha_{\mathrm{I}}}{\beta_{\mathrm{I}}}(\frac{\kappa}{\beta_{\mathrm{I}}})^{\alpha_{\mathrm{I}}-1}, \tag{162}$$

while the infection times follow a Weibullean distribution:

$$\psi_{\mathrm{inf}}(\kappa) = \frac{\alpha_{\mathrm{I}}}{\beta_{\mathrm{I}}}(\frac{\kappa}{\beta_{\mathrm{I}}})^{\alpha_{\mathrm{I}}-1}e^{-(\frac{\kappa}{\beta_{\mathrm{I}}})^{\alpha_{\mathrm{I}}}}. \tag{163}$$

For $\alpha_{\mathrm{I}} > 1$, $\omega_{\mathrm{inf}}(\kappa)$ increases with κ. A greater value of α_{I} results in faster growth of $\omega_{\mathrm{inf}}(\kappa)$ with κ. Since the age of an active edge cannot be zero after it transmits the disease, the value of the infection rate $\omega_{\mathrm{inf}}(\kappa)$ will keep increasing until the corresponding infected node recovers. Supplementary Fig. 10 shows an example of how the infection rate of an active edge increases sharply with κ. The persistent enhancement effect of infection rate with time makes it harder for the mean-field theory to capture the dynamical correlation on the active edge, generating deviations between the simulation result for $\alpha_{\mathrm{I}} = 4$ from the mean-field prediction.

SUPPLEMENTARY REFERENCES

[1] Liptaj, A. Higher order derivatives of the inverse function (2007).

[2] Van Mieghem, P. *Performance Analysis of Communications Networks and Systems* (Cambridge University Press, 2009).

[3] Anderson, R. M. & May, R. M. *Infectious Diseases of Humans: Dynamics and Control* (Oxford University Press, 1992).

[4] Pastor-Satorras, R. & Vespignani, A. Epidemic spreading in scale-free networks. *Phys. Rev. Lett.* **86**, 3200 (2001).

[5] Van Mieghem, P., Omic, J. & Kooij, R. Virus spread in networks. *IEEE/ACM Trans. Network. (TON)* **17**, 1–14 (2009).

[6] Van Mieghem, P. The n-intertwined SIS epidemic network model. *Computing* **93**, 147–169 (2011).

[7] Rand, D. Correlation equations and pair approximations for spatial ecologies. *Chapter 4 in Advanced Ecological Theory: Principles and Applications* **100** (1999).

[8] Keeling, M. J. The effects of local spatial structure on epidemiological invasions. *Proc. R. Soc. Lond. B* **266**, 859–867 (1999).

[9] Keeling, M. & Rand, D. Spatial correlations and local fluctuations in host-parasite models. *in From Finite to Infinite Dimensional Dynamical Systems* 5–57 (2001).

[10] Georgiou, N., Kiss, I. Z. & Scalas, E. Solvable non-Markovian dynamic network. *Phys. Rev. E* **92**, 042801 (2015).

[11] Kiss, I. Z., Röst, G. & Vizi, Z. Generalization of pairwise models to non-Markovian epidemics on networks. *Phys. Rev. Lett.* **115**, 078701 (2015).

[12] Sherborne, N., Miller, J. C., Blyuss, K. B. & Kiss, I. Z. Mean-field models for non-Markovian epidemics on networks. *J. Math. Biol.* **76**, 755–778 (2018).

[13] Eames, K. T. & Keeling, M. J. Modeling dynamic and network heterogeneities in the spread of sexually transmitted diseases. *Proc. Nat. Acad. Sci. (USA)* **99**, 13330–13335 (2002).

[14] Gleeson, J. P. High-accuracy approximation of binary-state dynamics on networks. *Phys. Rev. Lett.* **107**, 068701 (2011).

[15] Vazquez, A., Racz, B., Lukacs, A. & Barabasi, A.-L. Impact of non-Poissonian activity patterns on spreading processes. *Phys. Rev. Lett.* **98**, 158702 (2007).

[16] Min, B., Goh, K.-I. & Vazquez, A. Spreading dynamics following bursty human activity patterns. *Phys. Rev. E* **83**, 036102 (2011).

[17] Iribarren, J. L. & Moro, E. Impact of human activity patterns on the dynamics of information diffusion. *Phys. Rev. Lett.* **103**, 038702 (2009).

[18] Iribarren, J. L. & Moro, E. Branching dynamics of viral information spreading. *Phys. Rev. E* **84**, 046116 (2011).

[19] Fernández-Gracia, J., Eguíluz, V. M. & San Miguel, M. Update rules and interevent time distributions: Slow ordering versus no ordering in the voter model. *Phys. Rev. E* **84**, 015103 (2011).

[20] Watts, D. J. & Strogatz, S. H. Collective dynamics of small-world networks. *Nature* **393**, 440 (1998).

[21] Barthélemy, M., Barrat, A., Pastor-Satorras, R. & Vespignani, A. Velocity and hierarchical spread of epidemic outbreaks in scale-free networks. *Phys. Rev. Lett.* **92**, 178701 (2004).

[22] Pastor-Satorras, R. & Vespignani, A. Epidemic spreading in scale-free networks. *Phys. Rev. Lett.* **86**, 3200 (2001).

[23] Boguá, M., Pastor-Satorras, R. & Vespignani, A. Epidemic spreading in complex networks with degree correlations. In *Statistical mechanics of complex networks*, 127–147 (Springer, 2003).

[24] Onnela, J.-P. *et al.* Structure and tie strengths in mobile communication networks. *Proc. Nat. Acad. Sci. (USA)* **104**, 7332–7336 (2007).

[25] Park, Y., Moore, C. & Bader, J. S. Dynamic networks from hierarchical Bayesian graph clustering. *PloS one* **5**, e8118 (2010).

[26] Boguná, M., Lafuerza, L. F., Toral, R. & Serrano, M. Á. Simulating non-Markovian stochastic processes. *Phys. Rev. E* **90**, 042108 (2014).

[27] Van Mieghem, P. & van de Bovenkamp, R. Non-Markovian infection spread dramatically alters the susceptible-infected-susceptible epidemic threshold in networks. *Phys. Rev. Lett.* **110**, 108701 (2013).

[28] Catanzaro, M., Boguná, M. & Pastor-Satorras, R. Generation of uncorrelated random scale-free networks. *Phys. Rev. E* **71**, 027103 (2005).
