# Supplementary material for: Equivalence and its invalidation between non-Markovian and Markovian spreading dynamics on complex networks
Source: Nat Commun. 2019 Aug 23;10:3748. doi: 10.1038/s41467-019-11763-z (PMC6707263; doi:10.1038/s41467-019-11763-z)
Supplement: Supplementary file 3 — Source Data [file 41467_2019_11763_MOESM3_ESM.zip › data/README.pdf]

Folder Name: Figure_3

-This folder contains 6 files which record the data of Figure 3 in the main text.

file name	illustration
Fig3_I_Random.dat	Results with Type-I mechanism on the Random Network
Fig3_I_Scale-free.dat	Results with Type-I mechanism on the Scale-free Network
Fig3_I_Hamsterster.dat	Results with Type-I mechanism on the Hamsterster Network
Fig3_II_Random.dat	Results with Type-II mechanism on the Random Network
Fig3_II_Scale-free.dat	Results with Type-II mechanism on the Scale-free Network
Fig3_II_Hamsterster.dat	Results with Type-II mechanism on the Hamsterster Network

- In the 6 files, all **odd** columns show **abscissa** data, i.e., the values of $\boldsymbol{t}$, and all **even** columns show **ordinate** data, i.e., the values of $\boldsymbol{I(t)}$.
- All the data in the **odd** columns represent **independent variables**, while all the data in the **next columns**, i.e., the **even** columns, represent the **corresponding dependent variables**.
- In the 6 files, the **2nd, 6th, 10th, and 14th** columns show the **simulation** results with $\boldsymbol{\alpha_{\mathrm{I}} = 0.5, 1.0, 2.0, 4.0}$, respectively.
- In the 6 files, the **4th, 8th, 12th, and 16th** columns show the **theoretical** results with $\boldsymbol{\alpha_{\mathrm{I}} = 0.5, 1.0, 2.0, 4.0,}$ respectively.

Folder Name: Figure_4

-This folder contains 3 files which record the data of Figure 4 in the main text.

file name	illustration
Fig4_II_Random.dat	Results with Type-II mechanism on the Random Network
Fig4_II_Scale-free.dat	Results with Type-II mechanism on the Scale-free Network
Fig4_II_Hamsterster.dat	Results with Type-II mechanism on the Hamsterster Network

- In the 3 files, all **odd** columns show **abscissa** data, i.e., the values of $\boldsymbol{\lambda}_{\mathbf{eff}}$, and all **even** columns show **ordinate** data, i.e., the values of $\tilde{\boldsymbol{I}}$.

- All the data in the **odd** columns represent **independent variables**, while all the data in the **next** columns, i.e., the **even** columns, represent the **corresponding dependent variables**.

- In the 3 files, the **2nd, 4th, 6th and 8th** columns show the **simulation** results with $\boldsymbol{\alpha}_{\mathrm{I}} = \mathbf{0.5, 1.0, 2.0, 4.0}$, respectively. And the **10th and 12th** columns show the results of **Markovian Simulation** and **Markovian Theory**, respectively.

Folder Name: Figure_5

- This folder contains 3 files which record the data of Figure 5 in the main text.

file name	illustration
Fig5_I_Random.dat	Results with Type-I mechanism on the Random Network
Fig5_I_Scale-free.dat	Results with Type-I mechanism on the Scale-free Network
Fig5_I_Hamsterster.dat	Results with Type-I mechanism on the Hamsterster Network

- In the 3 files, all **odd** columns show **abscissa** data, i.e., the values of $\boldsymbol{\lambda}_{\mathbf{eff}}$, and all **even** columns show **ordinate** data, i.e., the values of $\tilde{\boldsymbol{I}}$.

- All the data in the **odd** columns represent **independent variables**, while all the data in the **next** columns, i.e., the **even** columns, represent the **corresponding dependent variables**.

- In the 3 files, the **2nd, 4th, 6th and 8th** columns show the **simulation** results with $\boldsymbol{\alpha}_{\mathbf{R}} = \mathbf{0.5, 1.0, 2.0, 4.0}$**,** respectively. And the **10th and 12th** columns show the results of **Markovian Simulation** and **Markovian Theory**, respectively.

Folder Name: Supplementary_Figure_2

- This folder contains 4 files which record the data of Supplementary Figure 2 in the Supplementary Information.

file name	illustration
sFig2_I_simulation_1st.dat	Results with Type-I mechanism on the Random Network
sFig2_II_simulation_1st.dat	Results with Type-II mechanism on the Random Network
sFig2_I_2nd.dat	Results with Type-I mechanism on the Random Network
sFig2_II_2nd.dat	Results with Type-II mechanism on the Random Network

- In the 4 files, all **odd** columns show **abscissa** data, i.e., the values of $\boldsymbol{t}$, and all **even** columns show **ordinate** data, i.e., the values of $\boldsymbol{I(t)}$.
- All the data in the **odd** columns represent **independent variables**, while all the data in the **next** columns, i.e., the **even** columns, represent the **corresponding dependent variables**.
- In the files, **sFig2_I_simulation_1st.dat** and **sFig2_II_simulation_1st.dat**, the **2nd, 6th, 10th, and 14th** columns show the **simulation** results with $\boldsymbol{\alpha_{\mathrm{I}} = 0.5, 1.0, 2.0, 4.0}$, respectively.
- In the files, **sFig2_I_simulation_1st.dat** and **sFig2_II_simulation_1st.dat**, the **4th, 8th, 12th, and 16th** columns show the results of **first-order mean field theory** with $\boldsymbol{\alpha_{\mathrm{I}} = 0.5, 1.0, 2.0, 4.0}$, respectively.
- In the files, **sFig2_I_2nd.dat** and **sFig2_II_2nd.dat**, the **2nd, 4th, 6th, and 8th** columns show the results of **second-order mean field theory** with $\boldsymbol{\alpha_{\mathrm{I}} = 0.5, 1.0, 2.0, 4.0}$**,** respectively.

Folder Name: Supplementary_Figure_3

- This folder contains 2 files which record the data of Supplementary Figure 3 in the Supplementary Information.

file name	illustration
sFig3_I.dat	Results with Type-I mechanism
sFig3_II.dat	Results with Type-II mechanism

-In the 2 files, all **odd** columns show **abscissa** data, i.e., the values of $\boldsymbol{\alpha}_{\mathrm{I}}$, and all **even** columns show **ordinate** data, i.e., the values of $\boldsymbol{T}_{\mathbf{half}}$.

- All the data in the **odd** columns represent **independent variables**, while all the data in the **next** columns, i.e., the **even** columns, represent the **corresponding dependent variables**.

-In the 2 files, the **2nd, 6th, and 10th** columns show the **simulation** results on **Random, Scale-free and Hamsterster** Networks, respectively.

–In the 2 files, the **4th, 8th, and 12th** columns show the **theoretical** results on **Random, Scale-free and Hamsterster** Networks, respectively.

Folder Name: Supplementary_Figure_4

- This folder contains 2 files which record the data of Supplementary Figure 4 in the Supplementary Information.

file name	illustration
sFig4_gamma.dat	Results with the argument γ
sFig4_D_low.dat	Results with the argument D_{low}

- In the file, **sFig4_gamma.dat**, all **odd** columns show **abscissa** data, i.e., the values of $\boldsymbol{\gamma}$, and all **even** columns show **ordinate** data, i.e., the values of $\boldsymbol{T}_{\mathbf{half}}$.
- In the file, **sFig4__D_low.dat**, all **odd** columns show **abscissa** data, i.e., the values of $\boldsymbol{D}_{\mathbf{low}}$, and all **even** columns show **ordinate** data, i.e., the values of $\boldsymbol{T}_{\mathbf{half}}$.
- All the data in the **odd** columns represent **independent variables**, while all the data in the **next** columns, i.e., the **even** columns, represent the **corresponding dependent variables**.
- In the 2 files, the **2nd and 6th** columns show the **simulation** results with **Type-I and Type-II** edge activation mechanisms, respectively.
– In the 2 files, the **4th and 8th** columns show the **theoretical** results with **Type-I and Type-II** edge activation mechanisms, respectively.

Folder Name: Supplementary_Figure_5

- This folder contains 12 files which record the data of Supplementary Figure 5 in the Supplementary Information.

file name	illustration
sFig5_I_Random_i.dat	Results with Type-I mechanism on the Random Network
sFig5_I_Scale-free_i.dat	Results with Type-I mechanism on the Scale-free Network
sFig5_I_Hamsterster_i.dat	Results with Type-I mechanism on the Hamsterster Network
sFig5_I_Random_s.dat	Results with Type-I mechanism on the Random Network
sFig5_I_Scale-free_s.dat	Results with Type-I mechanism on the Scale-free Network
sFig5_I_Hamsterster_s.dat	Results with Type-I mechanism on the Hamsterster Network
sFig5_II_Random_i.dat	Results with Type-II mechanism on the Random Network
sFig5_II_Scale-free_i.dat	Results with Type-II mechanism on the Scale-free Network
sFig5_II_Hamsterster_i.dat	Results with Type-II mechanism on the Hamsterster Network
sFig5_II_Random_s.dat	Results with Type-II mechanism on the Random Network
sFig5_II_Scale-free_s.dat	Results with Type-II mechanism on the Scale-free Network
sFig5_II_Hamsterster_s.dat	Results with Type-II mechanism on the Hamsterster Network

- In the files, **sFig5_I_Random_i.dat, sFig5_I_Scale-free_i.dat, sFig5_I_Hamsterster_i.dat, sFig5_II_Random_i.dat, sFig5_II_Scale-free_i.dat and sFig5_II_Hamsterster_i.dat (i.e., the files XXXX_i.dat),** all **odd** columns show **abscissa** data, i.e., the values of $\boldsymbol{\tau}$, and all **even** columns show **ordinate** data, i.e., the values of $\tilde{\boldsymbol{I}}(\boldsymbol{\tau})$.
- In the files, **sFig5_I_Random_s.dat, sFig5_I_Scale-free_sdat, sFig5_I_Hamsterster_s.dat, sFig5_II_Random_s.dat, sFig5_II_Scale-free_s.dat and sFig5_II_Hamsterster_s.dat (i.e., the files XXXX_s.dat)**, all **odd** columns show **abscissa** data, i.e., the values of $\boldsymbol{\tau}$, and all **even** columns show **ordinate** data, i.e., the values of $\tilde{\boldsymbol{S}}(\boldsymbol{\tau})$.
- All the data in the **odd** columns represent **independent variables**, while all the data in the **next** columns, i.e., the **even** columns, represent the **corresponding dependent variables**.
- In the files, **sFig5_I_Random_i.dat, sFig5_II_Random_i.dat, sFig5_I_Random_s.dat and sFig5_II_Random_s.dat (i.e., the files XX_Random_X.dat)**, the **2nd, 4th and 6th** columns show the **simulation** results with $\boldsymbol{\alpha}_{\mathrm{I}} = \mathbf{0.5, 1.0, 2.0}$, respectively.
- In the files, **sFig5_I_Random_i.dat, sFig5_II_Random_i.dat, sFig5_I_Random_s.dat and sFig5_II_Random_s.dat (i.e., the files XX_Random_X.dat)**, the **8th, 10th and 12th** columns show the **theoretical** results with $\boldsymbol{\alpha}_{\mathrm{I}} = \mathbf{0.5, 1.0, 2.0}$, respectively.
- In the files, **sFig5_I_Scale-free_i.dat, sFig5_II_Scale-free_i.dat, sFig5_I_Scale-free_s.dat, sFig5_II_Scale-free_s.dat, sFig5_I_Hamsterster_i.dat, sFig5_II_Hamsterster_i.dat, sFig5_I_Hamsterster_s.dat and sFig5_II_Hamsterster_s.dat (i.e., the files XX_Scale-free_X.dat and XX_Hamsterster_X.dat)**, the **2nd, 4th, 6th and 8th** columns show the **simulation** results with $\boldsymbol{\alpha}_{\mathrm{I}} = \mathbf{0.5, 1.0, 2.0, 4.0}$, respectively.
- In the files, **sFig5_I_Scale-free_i.dat, sFig5_II_Scale-free_i.dat, sFig5_I_Scale-free_s.dat, sFig5_II_Scale-free_s.dat, sFig5_I_Hamsterster_i.dat, sFig5_II_Hamsterster_i.dat, sFig5_I_Hamsterster_s.dat and sFig5_II_Hamsterster_s.dat (i.e., the files XX_Scale-free_X.dat and XX_Hamsterster_X.dat)**, the **10th, 12th, 14th and 16th** columns show the **theoretical** results with $\boldsymbol{\alpha}_{\mathrm{I}} = \mathbf{0.5, 1.0, 2.0, 4.0}$, respectively.

Folder Name: Supplementary_Figure_6

- This folder contains 3 files which record the data of Supplementary Figure 6 in the Supplementary Information.

file name	illustration
sFig6_I_Random.dat	Results with Type-I mechanism on the Random Network
sFig6_I_Scale-free.dat	Results with Type-I mechanism on the Scale-free Network
sFig6_I_Hamsterster.dat	Results with Type-I mechanism on the Hamsterster Network

- In the 3 files, all **odd** columns show **abscissa** data, i.e., the values of $\boldsymbol{\lambda}_{\mathbf{eff}}$, and all **even** columns show **ordinate** data, i.e., the values of $\tilde{\boldsymbol{I}}$.
- All the data in the **odd** columns represent **independent** variables, while all the data in the **next** columns, i.e., the **even** columns, represent the **corresponding dependent variables**.
- In the 3 files, the **2nd, 4th, 6th and 8th** columns show the **simulation** results with $\boldsymbol{\alpha}_{\mathrm{R}} = \mathbf{0.5, 1.0, 2.0, 4.0}$, respectively. And the **10th and 12th** columns show the results of **Markovian Simulation** and **Markovian Theory**, respectively.

Folder Name: Supplementary_Figure_7

-This folder contains 4 files which record the data of Supplementary Figure 7 in the Supplementary Information.

**file name**	**illustration**
sFig7_I_transient.dat	Results with Type-I mechanism in transient regime
sFig7_II_transient.dat	Results with Type-II mechanism in transient regime
sFig7_I_steady.dat	Results with Type-I mechanism in steady state
sFig7_II_steady.dat	Results with Type-II mechanism in steady state

- In the files, **sFig7_I_transient.dat and sFig7_II_transient.dat**, all **odd** columns show **abscissa** data, i.e., the values of $\boldsymbol{t}$, and all **even** columns show **ordinate** data, i.e., the values of $\boldsymbol{I(t)}$.
- In the files, **sFig7_I_steady.dat and sFig7_II_steady.dat**, all **odd** columns show **abscissa** data, i.e., the values of $\boldsymbol{\lambda}_{\mathbf{eff}}$, and all **even** columns show **ordinate** data, i.e., the values of $\tilde{\boldsymbol{I}}$.
- All the data in the **odd** columns represent **independent variables**, while all the data in the **next** columns, i.e., the **even** columns, represent the **corresponding dependent variables**.
- In the files, **sFig7_I_transient.dat and sFig7_II_transient.dat**, the **2nd, 6th, 10th, and 14th** columns show the **simulation** results with $\boldsymbol{\alpha}_{\mathrm{I}} = \mathbf{0.5, 1.0, 2.0, 4.0}$, respectively.
- In the files, **sFig7_I_transient.dat and sFig7_II_transient.dat**, the **4th, 8th, 12th, and 16th** columns show the **theoretical** results with $\boldsymbol{\alpha}_{\mathrm{I}} = \mathbf{0.5, 1.0, 2.0, 4.0,}$ respectively.
- In the files, **sFig7_I_steady.dat and sFig7_II_steady.dat**, the **2nd, 4th, 6th and 8th** columns show the **simulation** results with $\boldsymbol{\alpha}_{\mathrm{I}} = \mathbf{0.5, 1.0, 2.0, 4.0,}$ respectively. And the **10th and 12th** columns show the results of **Markovian Simulation** and **Markovian Theory**, respectively.

Folder Name: Supplementary_Figure_8

-This folder contains 3 files which record the data of Supplementary Figure 8 in the Supplementary Information.

file name	illustration
sFig8_III_Random.dat	Results with Type-III mechanism on the Random Network
sFig8_III_Scale-free.dat	Results with Type-III mechanism on the Scale-free Network
sFig8_III_Hamsterster.dat	Results with Type-III mechanism on the Hamsterster Network

- In the 3 files, all **odd** columns show **abscissa** data, i.e., the values of $\boldsymbol{t}$, and all **even** columns show **ordinate** data, i.e., the values of $\boldsymbol{I(t)}$.
- All the data in the **odd** columns represent **independent variables**, while all the data in the **next** columns, i.e., the **even** columns, represent the **corresponding dependent variables**.
- In the 3 files, the **2nd, 6th, 10th, and 14th** columns show the **simulation** results with $\boldsymbol{\alpha_{\mathrm{I}}} = \mathbf{0.5, 1.0, 2.0, 4.0}$, respectively.
- In the 3 files, the **4th, 8th, 12th, and 16th** columns show the **theoretical** results with $\boldsymbol{\alpha_{\mathrm{I}}} = \mathbf{0.5, 1.0, 2.0, 4.0,}$ respectively.

Folder Name: Supplementary_Figure_9

-This folder contains 3 files which record the data of Supplementary Figure 9 in the Supplementary Information.

file name	illustration
sFig9_III_Random.dat	Results with Type-III mechanism on the Random Network
sFig9_III_Scale-free.dat	Results with Type-III mechanism on the Scale-free Network
sFig9_III_Hamsterster.dat	Results with Type-III mechanism on the Hamsterster Network

- In the 3 files, all **odd** columns show **abscissa** data, i.e., the values of $\boldsymbol{\lambda}_{\mathbf{eff}}$, and all **even** columns show **ordinate** data, i.e., the values of $\tilde{\boldsymbol{I}}$.
- All the data in the **odd** columns represent **independent variables**, while all the data in the **next** columns, i.e., the **even** columns, represent the **corresponding dependent variables**.
- In the 3 files, the **2nd, 4th, 6th and 8th** columns show the **simulation** results with $\boldsymbol{\alpha}_{\mathbf{I}} = \mathbf{0.5, 1.0, 2.0, 4.0}$ **,** respectively. And the **10th and 12th** columns show the results of **Markovian Simulation** and **Markovian Theory**, respectively.
